# TGF-β signaling alters H4K20me3 status via miR-29 and contributes to cellular senescence and cardiac aging

**DOI:** 10.1038/s41467-018-04994-z

**Published:** 2018-07-02

**Authors:** Guoliang Lyu, Yiting Guan, Chao Zhang, Le Zong, Lei Sun, Xiaoke Huang, Li Huang, Lijun Zhang, Xiao-Li Tian, Zhongjun Zhou, Wei Tao

**Affiliations:** 1 The MOE Key Laboratory of Cell Proliferation and Differentiation, School of Life Sciences, Peking University, Beijing, 100871 China; 2 Department of Human Population Genetics, Human Aging Research Institute and School of Life Science, Nanchang University, Nanchang, 330031 China; 3 School of Biomedical Sciences, LKS Faculty of Medicine, The University of Hong Kong, 21 Sassoon Road, Hong Kong, China; 4 Shenzhen Institute of Innovation and Research, The University of Hong Kong, Nanshan, Shenzhen 518000 China

## Abstract

Cellular senescence is a well-orchestrated programmed process involved in age-related pathologies, tumor suppression and embryonic development. TGF-β/Smad is one of the predominant pathways that regulate damage-induced and developmentally programmed senescence. Here we show that canonical TGF-β signaling promotes senescence via miR-29-induced loss of H4K20me3. Mechanistically, oxidative stress triggers TGF-β signaling. Activated TGF-β signaling gives rise to acute accumulation of miR-29a and miR-29c, both of which directly suppress their novel target, Suv4-20h, thus reducing H4K20me3 abundance in a Smad-dependent manner, which compromises DNA damage repair and genome maintenance. Loss of H4K20me3 mediated by the senescent TGF-β/miR-29 pathway contributes to cardiac aging in vivo. Disruption of TGF-β signaling restores H4K20me3 and improves cardiac function in aged mice. Our study highlights the sequential mechanisms underlying the regulation of senescence, from senescence-inducing triggers to activation of responsive signaling followed by specific epigenetic alterations, shedding light on potential therapeutic interventions in cardiac aging.

## Abstract (web summary)

Cellular senescence is associated with epigenetic remodeling. Here, the authors report that TGF-β signaling promotes miR-29 mediated loss of H4K20me3 thus accelerating senescence.

## Introduction

Cellular senescence is featured by a state of cell proliferation arrest, accumulation of senescence-associated β-galactosidase (SA-β-gal)^1^, emergence of senescence-associated secretory phenotypes (SASP)^2^ and expression of cyclin-dependent kinase inhibitors (CKIs)^3,4^. Cellular senescence is triggered by a myriad of extracellular and intracellular stimuli^5–7^, and it is involved, pathologically or physiologically, in age-related disorders, tumor suppression and tissue patterning^8–10^. The process of senescence is coordinated through a variety of regulatory networks directed by endogenous and exogenous senescence-inducing signals^11,12^.

TGF-β/Smad signaling is one of the prominent pathways regulating both damage-induced senescence and developmentally programmed senescence^13^. The pathway is evolutionarily conserved and participates in an enormous range of biological processes that influence various physiological activities, including cell cycle control, wound healing, bone morphogenesis, carcinogenesis, tumor suppression and differentiation in cell-type specific and context-dependent manners^14–16^. Upon binding of TGF-β, the type II (TβRII) and type I receptor (TβRI) kinases undergo a series of complex formation and phosphorylation events, leading to activation of TβRI, followed by signal transduction via the formation of complexes comprising phosphorylated receptor-regulated pSmad2/3 and the common mediator Smad4. Next, the Smad complex is translocated into the nucleus, where it regulates transcription of downstream target genes through physical interaction and functional cooperation with other co-factors^17^. In vivo and in vitro models have confirmed that TGF-β signaling, activated by SASP or other developmental cues, regulates senescence via up-regulation of p15 and/or p21 in damage-induced and developmental senescence^18^. These findings indicate that TGF-β pathway plays an important role in senescence as one of the key sensors mediating senescent signaling in response to environmental stresses and endogenous signals.

Epigenetic programing in the forms of DNA methylation patterns^19,20^, histone modification landscapes^21–23^, chromatin architecture organization^24,25^ and non-coding RNAs^26,27^ contributes to senescence and is widely accepted as a hallmark of senescence. Epigenetic alterations influence senescence by impinging on DNA damage repair, telomere length and metabolic pathways^28^, or activating expression of senescence-related genes and miRNAs^29,30^. Multiple lines of evidence suggest that alterations of chromatin states are closely linked to the control of cellular senescence^31–33^. Cells can sense diverse senescence-inducing stimuli, which activate signaling pathways that drive changes in chromatin status^34,35^. However, the pathways through which senescence signals cause such alterations remain largely unknown.

In this study, we report that TGF-β/Smad signaling triggers miR-29-mediated reduction of H4K20me3 abundance, which promotes cellular senescence. We demonstrate a sequential regulatory axis, in which H4K20me3, as a responsive downstream epigenetic effector of the TGF-β/Smad pathway, is negatively regulated by miR-29 to regulate cellular senescence. Disruption of TGF-β signaling restores global H4K20me3 abundance in the aged murine heart and partially improves cardiac function in vitro. Our study reveals an epigenetics-based responsive pathway that drives alterations in histone modification status during cellular senescence.

## Results

### miR-29-mediated loss of H4K20me3 promotes senescence

**Fig. 1:**
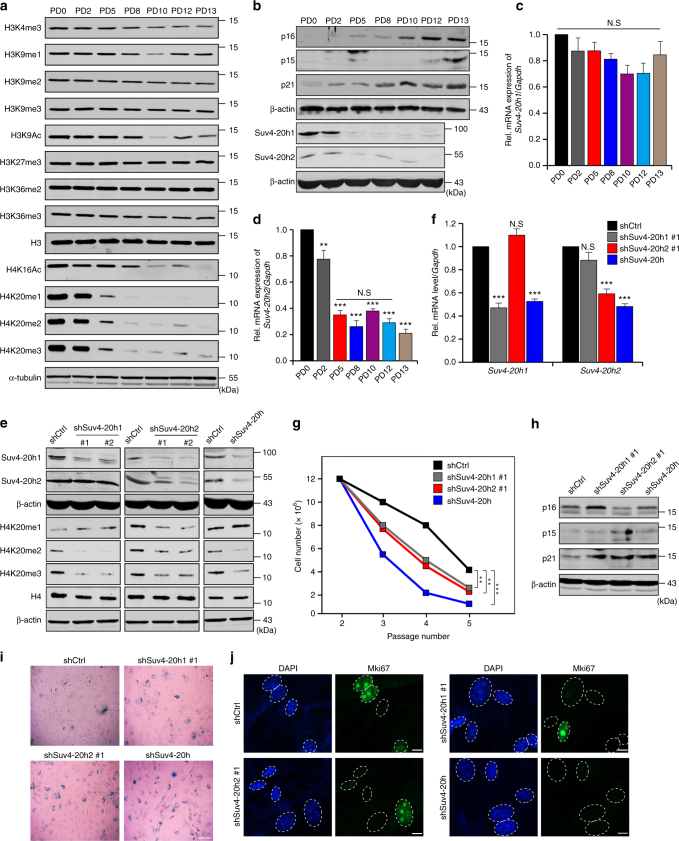
Loss of H4K20me3 promotes cellular senescence. **a** Western blot for total protein of the indicated histone modifications from serially passaged MEFs. H3, H4 and α-tubulin served as loading controls. **b** Western blots showing expression of p16, p15, p21, Suv4-20h1 and Suv4-20h2 during MEFs senescence. β-actin served as a loading control. **c**, **d** Real-time qPCR analysis of *Suv4-20h1* (**c**) and *Suv4-20h2* (**d**) mRNA expression in different passages of MEFs. **e**, **f** Western blot (**e**) and RT-qPCR (**f**) for Suv4-20h in knockdown cells. **g** Growth curve of Suv4-20h-depleted cells. **h** Expression of p16, p15 and p21 in Suv4-20h knockdown cells. β-actin served as a loading control. **i** SA-β-gal staining in control (Ctrl) and Suv4-20h (shSuv4-20h) knockdown cells. Scale bar, 20 μm. **j** Immunofluorescent staining of Mki67 in control (Ctrl) and Suv4-20h depleted cells. Scale bar, 5 μm. Two-tailed unpaired Student *t*-tests were performed, **p < 0.01, ***p < 0.001

To examine senescence-associated changes of histone modifications, we performed histone modifications scanning in senescent cells. The results showed that H4K20me1, -me2, and -me3 exhibited prominent down-regulation in senescent mouse embryonic fibroblasts (MEFs) (Fig. 1a; Supplementary Fig. 1a–c). Accordingly, the expression levels of Suv4-20h1 and Suv4-20h2, the two major methyltransferases mediating H4K20me3, were also decreased during senescence (Fig. 1b–d). Furthermore, depletion of Suv4-20h1, Suv4-20h2 or both (designated as Suv4-20h) by shRNAs and treatment with selective Suv4-20h inhibitor A-196^36^ led to reduced H4K20me3 protein abundance and premature senescence (Fig. 1e–j and Supplementary Fig. 1d–k), consistent with a previous finding that knockout of *Suv4-20h1* and *Suv4-20h2* contributed to defective proliferation and cell cycle progression in MEFs^37^. These results suggest that H4K20me3 is a negative regulator of senescence.

**Fig. 2:**
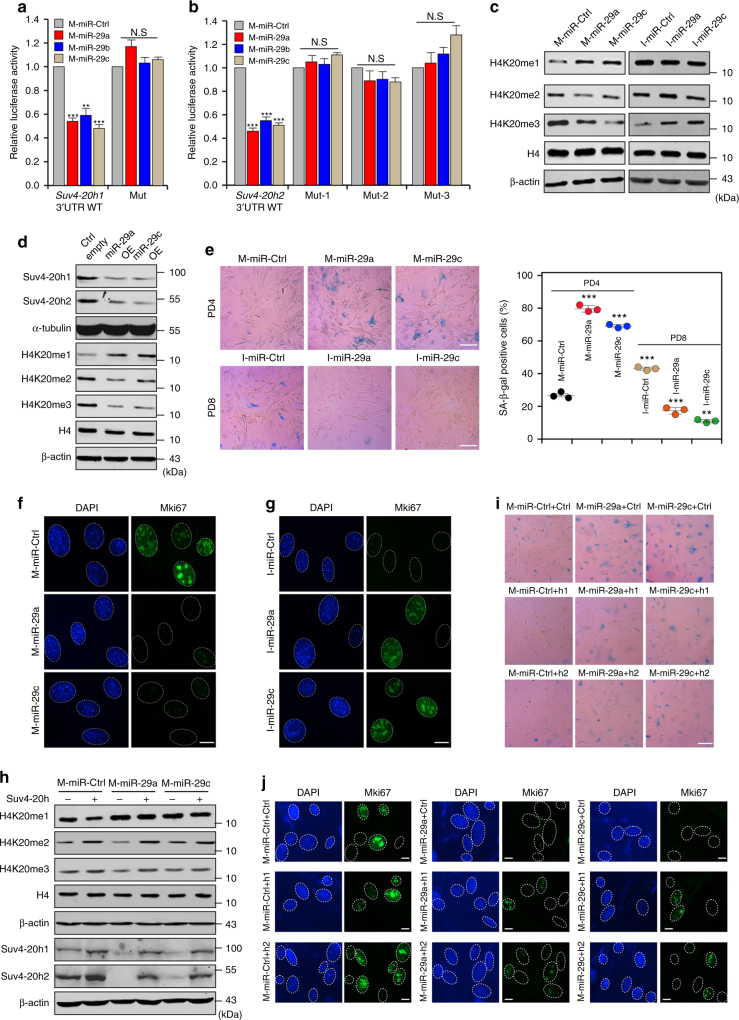
miR-29-mediated reduction of H4K20me3 leads to premature cellular senescence. **a**, **b** Luciferase assays with the wild-type *Suv4-20h1* 3′ UTR or mutated *Suv4-20h1* 3′ UTR (**a**), as well as with the wild-type *Suv4-20h2* 3′ UTR or mutated *Suv4-20h2* 3′ UTR (**b**) in the predicted binding site of miR-29a, miR-29b and miR-29c, transfected with control miRNA mimics (M-miR-Ctrl) or miR-29 mimics (M-miR-29a, M-miR-29b and M-miR-29c). **c** Protein levels of H4K20 were measured by western blotting in MEFs transfected with M-miR-29 or miR-29 inhibitors (I-miR-29a and I-miR-29c). β-actin served as a loading control. **d** Western blot for global H4K20 methylation in cells with lentivirus-mediated ectopic expression of the indicated miR-29. β-actin served as a loading control. **e** SA-β-gal staining and diagrams showing MEFs from PD4 transfected with M-miR-29 and MEFs from PD8 transfected with I-miR-29. Scale bar, 20 μm. One-way ANOVA with Dunnett’s multiple comparison test was performed. **f**, **g** Immunofluorescent staining of Mki67 in MEFs transfected with M-miR-29 (**f**) or I-miR-29 (**g**). Scale bar, 5 μm. **h** Cells were first transfected with M-miR-Ctrl, M-miR-29a or M-miR-29c, after which they were infected with pBabe, pBabe-Suv4-20h1 or pBabe-Suv4-20h2 and subjected to western blotting for the indicated protein. β-actin served as a loading control. **i** SA-β-gal staining analysis was performed as described for cells in **h**. Scale bar, 20 μm. **j** Immunofluorescent staining of Mki67 in the cells from **i**. Scale bar, 5 μm. The error bars show the s.d obtained from triplicate independent experiments. Two-tailed unpaired Student’s *t*-tests were performed, **p < 0.01, ***p < 0.001

Next, we looked into the mechanisms underlying the cellular alterations described above. miRNAs can silence gene expression via mRNA destabilization and/or translational repression^38^, so we reasoned that Suv4-20h silencing might be caused by miRNAs. Through bioinformatics analyses via TargetScan, PicTar and miRDB^39–41^, we identified three putative miRNAs targeting miRNA response elements (MREs) residing in 3′ untranslated region (3′ UTR) of *Suv4-20h1* mRNA: miR-29a-3p, miR-29b-3p and miR-29c-3p (denoted as miR-29a, miR-29b and miR-29c, respectively). The three miRNAs belong to the miR-29 family and function in a synergistic manner in a wide range of physiological activities^42–44^. It is likely that miR-29a, miR-29b and miR-29c target the 3′ UTRs of *Suv4-20h1* and *Suv4-20h2* via incomplete complementary binding to their potential MREs (Supplementary Fig. 2a). Apart from miR-29, three other miRNAs, miR-125a-5p, miR-125b-5p and miR-24-3p, were predicted to target the 3′ UTR of *Suv4-20h2* (Supplementary Fig. 2b, c). To validate whether Suv4-20h1 and Suv4-20h2 are bona fide targets of the predicted miRNAs, we generated luciferase reporter constructs, in which the 3′ UTR of *Suv4-20h* was placed behind the luciferase gene. As shown in Supplementary Fig. 2d,e, the luciferase activity assay indicated that miR-29 targeted the 3′ UTR of *Suv4-20h*, but miR-125a-3p, miR-125b-3p and miR-24-3p did not. The inhibitory effects were attenuated when the miR-29 binding sites on the MREs were mutated (Fig. 2a, b). Furthermore, forced expression or inhibition of miR-29, by individual transfection of miRNA mimics (M-miR-29) or inhibitors (I-miR-29a and I-miR-29c), resulted in decreased or increased H4K20me3 abundance, respectively (Fig. 2c and Supplementary Fig. 2f–l). Gain of function of miR-29 via lentiviral infection led to reduced protein levels of Suv4-20h and H4K20me3 (Fig. 2d and Supplementary Fig. 2m). These results indicate that Suv4-20h1 and Suv4-20h2 are novel direct targets of miR-29.

To investigate the role of miR-29 in senescence, we performed differential miRNA expression analyses in serially passaged MEFs, which revealed accumulation of miR-29a and miR-29c, but nearly unaltered abundance of miR-29b, in senescent cells (Supplementary Fig. 2n, o). Increased abundance of miR-29 (represented miR-29a and miR-29c hereinafter) in early passaged cells (PD4) accelerated senescence whereas decreased amounts of miR-29 in late passaged cells (PD8) delayed senescent phenotypes (Fig. 2e–g and Supplementary Fig. 2p). Moreover, re-introducing 3′ UTR-depleted *Suv4-20h1* and *Suv4-20h2* alleviated the miR-29-induced reduction in H4K20me3 abundance (Fig. 2h and Supplementary Fig. 2q, r) and inhibition of senescence progression (Fig. 2i, j and Supplementary Fig. 2s). Taken together, these results suggest that miR-29 directly suppresses Suv4-20h through incomplete complementarity to MREs in the 3′ UTR of *Suv4-20h* to promote cellular senescence.

### TGF-β signaling regulates expression of miR-29

**Fig. 3:**
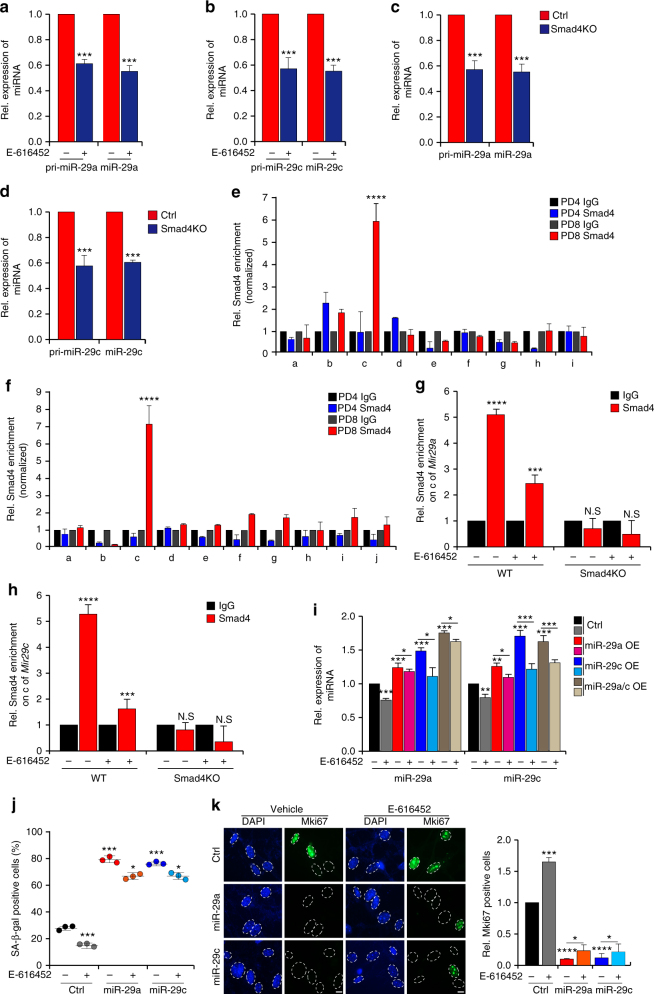
TGF-β signaling regulates miR-29 expression in a Smad-dependent manner during MEFs senescence. **a**–**d** Quantitative real-time PCR for pri-miR-29 and miR-29 in MEFs incubated with E-616452 (**a**, **b**) or *Smad4* knockout (Smad4KO) MEFs (**c**, **d**). **e**, **f** Chromatin immunoprecipitation-qPCR (ChIP-qPCR) assays of Smad4 from PD4 and PD8 MEFs. The *x*-axis represents the primer positons of the predicted promoters of Mir29a (**e**) and Mir29c (**f**). **g**, **h** Smad4 enrichment on *Mir29a* (**g**) and *Mir29c* (**h**) was measured in inhibitor-treated MEFs and Smad4KO MEFs. **i** MEFs with lentivirus-mediated expression of miR-29 were incubated with E-616452. Real-time PCR was used to measure miR-29 expression. **j** Diagrams showing SA-β-gal staining of MEFs collected from **i**. **k** Immunofluorescent staining showing the Mki67 signals in MEFs with the same treatment as those shown in **i**. Scale bar, 5 μm. The error bars represent the s.d obtained from triplicate independent experiments. Two-tailed unpaired Student’s *t*-tests were performed, **p < 0.01, ***p < 0.001

Next we explored the manner in which miR-29 expression is regulated during cellular senescence. The TGF-β/Smad pathway participates in the regulation of transcription or processing of miRNA in human/mouse endothelial cells^45^ and human primary pulmonary artery smooth muscle cells (PASMCs)^46^. We examined whether TGF-β signaling regulates biogenesis of miR-29 during MEFs senescence. Levels of primary miR-29 (pri-miR-29) and mature miR-29 were measured when canonical TGF-β signaling was either activated by TGF-β or abolished by E-616452, a TGF-β signaling inhibitor. Expression levels of pri-miR-29 and miR-29 were elevated upon activation of TGF-β signaling (Supplementary Fig. 3a), whereas their levels were decreased in the presence of E-616452 (Fig. 3a, b), suggesting that TGF-β signaling positively regulates transcription of miR-29.

Next, we assessed the impact of Smad4, a common downstream effector of TGF-β signaling, on transcription of *Mir29a* and *Mir29c*. Knockout or knockdown of *Smad4* (Smad4KO or shSmad4) reduced the abundance of pri-miR-29 and miR-29 (Fig. 3c, d and Supplementary Fig. 3b–e). Promoter luciferase reporter assays indicated increased luciferase activity in cells with ectopic expression of Smad4 and the fused reporter constructs containing predicted promoters of *Mir29a/c* upstream of the luciferase gene, in comparison with cells treated with the control vector (Supplementary Fig. 3f). Chromatin immunoprecipitation (ChIP) with Smad4 antibodies followed by qPCR indicated that Smad4 directly bound to upstream sequences harboring the Smad binding elements (SBEs) of *Mir29a* and *Mir29c* (Fig. 3e, f and Supplementary Fig. 3g, h). In addition, suppressing TGF-β signaling by inhibitor treatment or *Smad4* knockout impaired Smad4 enrichment on the SBEs of *Mir29a* and *Mir29c* (Fig. 3g, h), which suggests that Smad4 is involved in TGF-β-regulated transcriptional up-regulation of miR-29 in senescent MEFs. Meanwhile, disruption of the TGF-β pathway by E-616452 treatment mitigated the increase in miR-29 abundance (Fig. 3i and Supplementary Fig. 3i) and progression of senescence in cells with ectopic miR-29 (Fig. 3j, k and Supplementary Fig. 3j). Together, these results demonstrate that TGF-β signaling activates miR-29 expression in a Smad-dependent manner.

### TGF-β signaling accelerates senescence via miR-29/H4K20me3

**Fig. 4:**
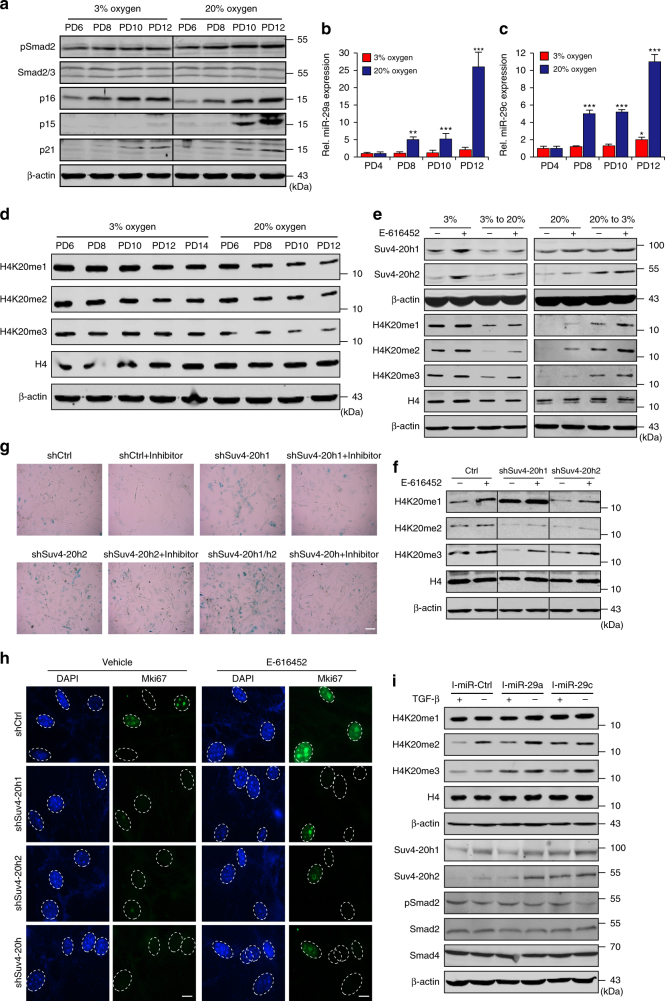
Regulation of senescence by TGF-β signaling is dependent on miR-29-induced inhibition of H4K20me3. **a** Western blot for the indicated proteins in MEFs grown in 3% oxygen or 20% oxygen. β-actin served as a loading control. **b**, **c** RT-qPCR analysis of miR-29 expression in the cells from **a**. **d** Western blot to assess total H4K20 methylation in serially passaged MEFs grown in 3% oxygen or 20% oxygen. β-actin served as a loading control. **e** MEFs grown in 3% oxygen and transferred to 20% oxygen (left) or MEFs grown in 20% oxygen and transferred to 3% oxygen (right) were treated with or without inhibitors. Protein changes were tested by western blot. H4 and β-actin served as loading controls. **f** Western blots showing altered H4K20 methylation in MEFs incubated with or without inhibitors upon knockdown of shCtrl, Suv4-20h1, Suv4-20h2 or both. **g** SA-β-gal staining showing alterations in the SA-β-gal signal in the cells from **f**. Scale bar, 20 μm. One-way ANOVA with Dunnett’s multiple comparison test analysis was performed. **h** Immunofluorescent staining showing the Mki67 signal in MEFs exposed to the same treatment as those shown in **f**. Scale bar, 5 μm. **i** Western blot for analyzing the indicated proteins in MEFs subjected to inhibition of miR-29 expression followed by TGF-β treatment. H4 and β-actin served as loading controls. The error bars represent the s.d. obtained from triplicate independent experiments. Two-tailed unpaired Student’s *t*-tests were performed, **p < 0.01, ***p < 0.001

Given that TGF-β signaling controls expression of miR-29, which represses H4K20me3 by inhibiting Suv4-20h, we tested whether activated TGF-β signaling expedites senescence by reducing the abundance of H4K20me3. We cultured cells in physiological (3%) oxygen (physioxia) and atmospheric (20%) oxygen (relative hyperoxia), because previous findings revealed that hyperoxia-induced oxidative stress activated the TGF-β pathway and accelerated senescence^47,48^. Indeed, compared to physioxia, hyperoxia promoted senescence and triggered activation of TGF-β signaling (Supplementary Fig. 4 and Fig. 4a). Activation of TGF-β signaling by oxidative stress was accompanied by increased miR-29 accumulation (Fig. 4b, c) and accelerated reduction in the abundance of total H4K20me3 (Fig. 4d).

Furthermore, inhibition of TGF-β signaling by inhibitor treatment and Smad4 depletion diminished miR-29 expression, attenuated the reduction in Suv4-20h1 and Suv4-20h2 expression and produced partial recovery of H4K20me3 (Fig. 4e and Supplementary Fig. 5a–c). Moreover, alterations in TGF-β signaling changed the progression of senescence and specifically altered H4K20me3 without affecting other histone modifications (Supplementary Fig. 5d–h). Disruption of TGF-β signaling by E-616452 restored H4K20me3 levels (Fig. 4f), attenuated SA-β-gal staining and enhanced the Mki67 signals (Fig. 4g, h) in Suv4-20h-depleted cells, suggesting that H4K20me3 is a downstream effector of TGF-β signaling. In addition, after miR-29 was destroyed by miRNA inhibitors, activation of TGF-β signaling did not down-regulate Suv4-20h and H4K20me3 (Fig. 4i). Collectively, these data demonstrate that TGF-β accelerates cellular senescence by facilitating the miR-29-mediated loss of H4K20me3.

### Reduction of H4K20me3 causes defective DNA damage repair

**Fig. 5:**
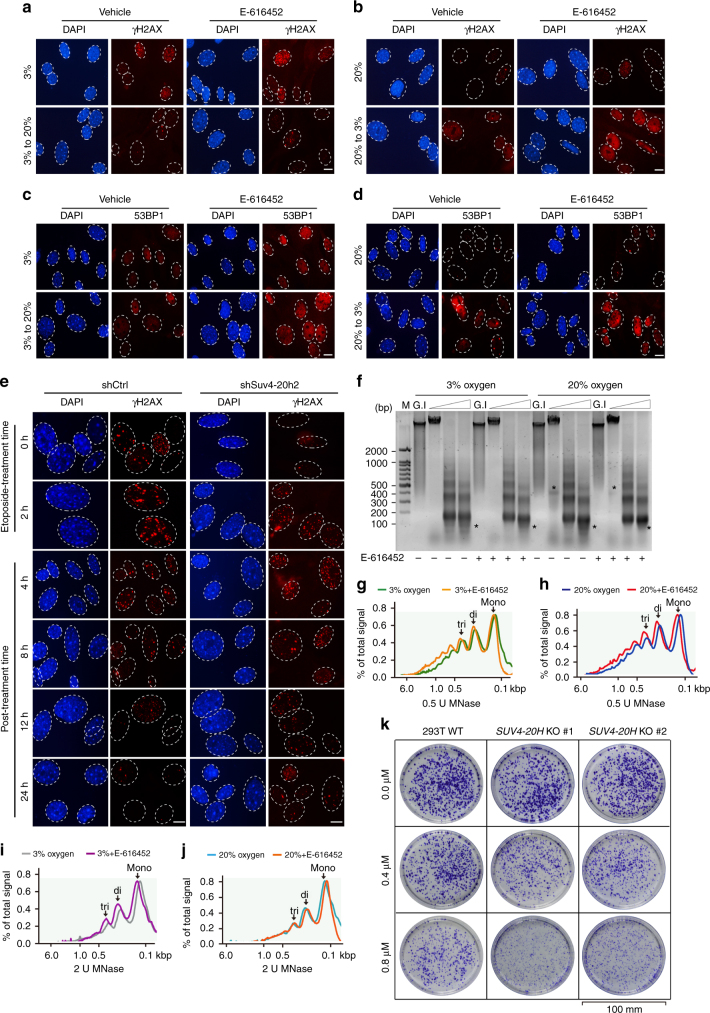
H4K20me3 contributes to DNA damage repair and genome maintenance. **a**–**d** MEFs cultured under the indicated conditions and treated with or without inhibitors were collected for immunofluorescent staining of γH2AX (**a**, **b**) or 53BP1 (**c**, **d**). Scale bar, 5 μm. **e** Etoposide-treated control (shCtrl) or *Suv4-20h2* knockdown (shSuv4-20h2) cells were subjected to γH2AX staining according to the indicated time course. Scale bar, 5 μm. **f**–**j** Nuclei from cells grown in 3% oxygen or 20% oxygen and treated with or without E-616452, were incubated with 0, 0.5 or 2 U of MNase for 5 min followed by DNA extraction, agarose gel electrophoresis and ethidium bromide staining. The asterisks indicate prominent fragment changes (**f**). The band densities were quantified using Image J software and illustrated as percentage of the signal subtracted background of the entire line from top to the bottom. Calibrated kilobase pair (kbp) sizes are indicated (**g**–**j**). **k** Wild-type HEK293T (293T WT) and *SUV4-20H* knockout cells (*SUV4-20H* KO #1 and *SUV4-20H* KO #2) were treated with the indicated concentration of etoposide, followed by crystal violet staining. Scale bar, 100 mm

H4K20me3 contributes to the processes of DNA damage repair and genomic maintenance^49–51^. We evaluated the role of TGF-β-miR-29 axis-regulated inhibition of H4K20me3 and DNA damage repair in senescent process. Inhibition of TGF-β signaling increased the fluorescent signals and/or protein levels of γH2AX and 53BP1 (Fig. 5a–d and Supplementary Fig. 6a–e), indicating that TGF-β signaling suppresses DNA damage repair in senescent cells. To investigate whether compromised DNA damage repair is a consequence of the global loss of H4K20me3, acute DNA damage was introduced by etoposide treatment for two hours. DNA damage repair was examined by measuring γH2AX and 53BP1 signals on the indicated time-course after the etoposide treatment was ceased. Suv4-20h2-depleted and Suv4-20h inhibitor-treated cells showed decreased global γH2AX protein abundance, prolonged persistence of γH2AX level and severe loss of 53BP1 after etoposide treatment (Fig. 5e and Supplementary Fig. 6f–h). These results indicate that H4K20me3 protects DNA from damage by facilitating DNA repair involving 53BP1.

To further substantiate whether TGF-β signaling compromises DNA damage repair and genomic maintenance by reducing the abundance of H4K20me3, the chromatin structures of normal cells and Suv4-20h knockdown cells, were analyzed by micrococcal nuclease (MNase) sensitivity assays in the presence and absence of E-616452. Activation of TGF-β signaling increased MNase sensitivity, while inhibition of TGF-β signaling decreased MNase accessibility (Fig. 5f–j). Moreover, inhibition of TGF-β signaling moderately attenuated MNase sensitivity in Suv4-20h-depleted cells (Supplementary Fig. 6i–k). The clonogenic cell survival assays utilizing HEK293T cells demonstrated that losing *SUV4-20H* conferred increased sensitivity to DNA damage because the abundance of H4K20me3 was reduced (Fig. 5k and Supplementary Fig. 6l, m). Taken together, these results suggest that TGF-β signaling delays DNA damage repair and impairs genomic maintenance by ablating H4K20me3.

### TGF-β/miR-29 signaling is involved in cardiac dysfunctions

**Fig. 6:**
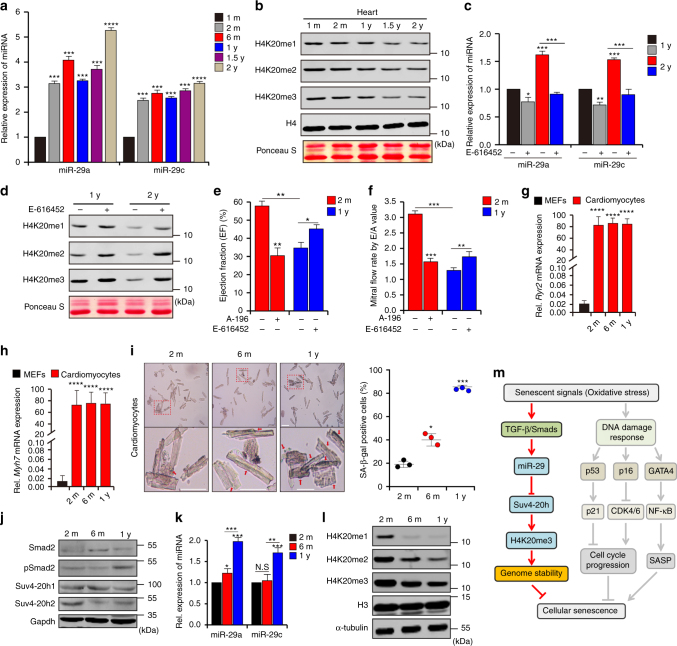
TGF-β/miR-29/H4K20me3 is involved in aging-associated cardiac dysfunction. **a** RT-qPCR to measure miR-29 expression, normalized to U6, in murine hearts (*n* = 4 per age) of the indicated ages (1-month-old, 1 m; 2-month-old, 2 m; 1-year-old, 1 y; 1.5-year-old, 1.5 y; 2-year-old, 2 y). **b** Western blot for H4K20 methylation in hearts of the indicated ages. **c** RT-qPCR to examine miR-29 expression in hearts of 1 y and 2 y mice, fed with DMSO or E-616452. The error bars represent the s.d obtained from triplicate independent experiments. **d** Western blot for H4K20 methylation in hearts from **c**. **e**, **f** Mice (2 m) fed with DMSO (*n* = 4) or A-196 (*n* = 4) and mice (1 y) treated with DMSO (*n* = 8) or E-616452 (*n* = 8) were subjected to cardiac function analysis using an echocardiographic imaging system (VisualSonics Vevo 2100, USA). EF (**e**) represents the ejection fraction of left ventricular flow, whereas E/A (**f**), the peak early filling (E-wave)/late diastolic filling (A-wave), represents the mitral flow rate. Representative images are shown in Supplementary Fig. 7e, f. The error bars represent the s.d. of the mean values. **g**, **h** RT-qPCR analysis of *Ryr2* (**g**) and *Myh7* (**h**) expression in cardiomyocytes isolated from mice of the indicated ages. **i** SA-β-gal staining of cardiomyocytes from mice as in **g**. The lower panel shows the magnified cells enclosed by red rectangular boxes in the images in the upper panel. Scale bar = 20 μm. The plot shows the proportion of SA-β-gal-stained cells among the different age groups. **j** Western blot for the indicated proteins in cells from **i**. **k** RT-qPCR for miR-29 expression in isolated cells from **i**. **l** Western blot for H4K20 methylations in cells from **f**. **m** Mechanisms proposed in this study. When cells undergo senescence-inducing stimuli, such as oxidative stress, TGF-β signaling is activated and serves as the signal transducer that promotes acute accumulation of miR-29, which in turn results in suppression of Suv4-20h and reduction in H4K20me3 abundance, leading to defective DNA damage repair and impaired maintenance of genome stability, and thus accelerating cellular senescence. Gapdh, H3 and α-tubulin were loading controls. Two-tailed unpaired Student’s *t*-tests, *p < 0.05, **p < 0.01, ***p < 0.001

Given that cellular senescence may accelerate aging when tissues exhaust their regenerative capacity^52–54^, and the TGF-β/Smad pathway participates in programmed senescence during tissue development, we investigated whether TGF-β-miR-29 signaling accelerates aging in vivo. We collected tissues samples (heart, liver, spleen, lung and kidney) from mice at different ages and found no significant change in the relative amount of H4K20m3 in the spleen or lung tissues as the age of the mice increased, although there was a slight increase in the relative amount of H4K20m3 in the kidney (Supplementary Fig. 7a). However, the older mice exhibited accumulation of cardiac miR-29 (Fig. 6a) and steady reduction in the abundance of H4K20 methylation (Fig. 6b). In addition, fluctuation in the abundance of H4K20me3 was observed in the liver; H4K20me3 abundance increased from1 month of age to 6 months of age, after which it was decreased in mice aged 1 year and 2 years (Supplementary Fig. 7b). After the mice were fed with E-616452 to inhibit TGF-β signaling, decreased H4K20me3 abundance was observed in the livers of 1-year-old mice, but a substantial increase was observed in the livers of 2-year-old mice (Supplementary Fig. 7c). These results suggest that modulation of H4K20me3 by TGF-β signaling might be involved in cardiac aging, probably because the cardiovascular system consumes a relatively large amount of oxygen and therefore it is likely to be more susceptible than other tissues to hyperoxic stress. Indeed, inhibition of TGF-β signaling led to down-regulation of cardiac miR-29 (Fig. 6c), concomitant with restoration of H4K20me3 (Fig. 6d).

We applied echocardiographic imaging to analyze the left ventricular function of young (2-month-old, 2m) mice treated with DMSO or Suv4-20h inhibitor A-196 and old (1-year-old, 1 y) mice fed with DMSO or TβRI inhibitor E-616452. In young mice, imaging analysis under B-mode, M-mode and pulsed wave Doppler-mode showed that A-196 treatment reduced the abundance of H4K20me3 (Supplementary Fig. 7d), weakened muscular movement of the left ventricle, decreased mitral flow and impaired systolic/diastolic function (Fig. 6e, f; Supplementary Fig. 7e and Supplementary Table 1), suggesting that H4K20me3 might be involved in protecting cardiac function. Treatment with E-616452 enhanced muscular movement of the left ventricle and partially rehabilitated mitral flow in old mice (Fig. 6e, f; Supplementary Fig. 7f and Supplementary Table 1), implying that inhibition of TGF-β signaling can contribute to preventing the age-associated declines in cardiac function. To validate the role of the TGF-β/miR-29 axis in regulating cardiac function, we isolated cardiomyocytes from adult mice aged 2 months (2 m), 6 months (6 m), and 1 year (1 y) (Fig. 6g, h). The cardiomyocytes of the 1 y adult mice showed increased SA-β-gal staining, activated TGF-β signaling, increased miR-29 accumulation and reduced abundance of H4K20me3 (Fig. 6i–l). Taken together, these results suggest that TGF-β/miR-29 signaling may contribute to age-associated cardiac dysfunction in vivo.

## Discussion

Our results demonstrate that H4K20me3, serving as a responsive epigenetic target of canonical TGF-β signaling, is negatively regulated by miR-29 during the progression of senescence, followed by specific epigenetic alterations. Thus, our findings illustrate a sequential regulatory network of cellular senescence, in which external stimuli (e.g., atmospheric hyperoxia) trigger accelerated up-regulation of TGF-β signaling, and the subsequent initiation and progression of senescence are dependent on miR-29-modulated dysregulation of H4K20me3. Therefore, senescence is regulated on the epigenetic level downstream of TGF-β receptor activation (Fig. 6m).

miRNAs are highly conserved single-stranded, non-coding RNA molecules of 18–25 nucleotides in length, which are involved in post-transcriptionally governing expression of their targets in a myriad of physiological processes including embryonic development^55^, immune responses^56,57^, cellular senescence and aging^58^. In recent years, miRNAs have emerged as crucial regulators of age-related alterations in organ function^59^, and the function of the cardiovascular system in particular^42,43,60^. For example, age-induced expression of miR-34a and inhibition of its target PNUTS act as a key mechanism that regulates cardiac contractile function during aging and after acute myocardial infarction. In another study, miR-29 was found to be regulated by acute myocardial infarction (MI) in the region of the heart adjacent to the infarct, and TGF-β signaling decreased expression of miR-29 after MI^42^. Some researchers have reported that increased miR-29 expression is closely associated with cardiac aging in aged mice and killifish^61,62^. These findings indicate that aging and other pathological contexts determine the regulatory response pattern of miR-29 downstream of TGF-β signaling. Here we found senescence- and aging-dependent miR-29 expression in fibroblasts and cardiomyocytes, and we established a potential link between miR-29 and TGF-β signaling. Therefore, it is reasonable to speculate that increased miR-29 expression might be a hallmark of cellular senescence and aging, and manipulation of the TGF-β/miR-29 pathway may provide opportunities for therapeutic modulation of aging and improvement of cardiac function.

It is noteworthy that, in contrast to our findings in MEFs, the abundance of H4K20me3 was found to be increased in prematurely senescent HGPS (Hutchinson–Gilford Progeria Syndrome) cells^63^, replicative and oncogene-induced senescent human diploid IMR90 fibroblasts^64^, and *Zmpste24* deficiency-mediated early senescent MEFs in culture^65^. Since TGF-β signaling is dynamically regulated in oncogene-induced and etoposide-induced senescence of IMR90 cells^18^, the seemingly paradoxical alterations of H4K20me3 abundance in models of senescence may be due to cell-type-specific and context-dependent TGF-β signaling. Indeed, H4K20me3 abundance was found to be increased in aged rat livers^66^, while aged murine cardiomyocytes, human umbilical vein endothelial cells (HUVECs) and human embryonic fibroblasts (HEFs) exhibited senescent phenotypic alterations of H4K20me3 similar to that of MEFs (Supplementary Fig. 8a–d and Supplementary Fig. 9). In addition, abolition of TGF-β signaling inhibited miR-29 expression and restored H4K20me3 abundance in the heart, but not in the kidney, lung and spleen, which indicated that the cell and tissue contexts determine the specific response to TGF-β during senescence and aging. Therefore, we propose that a loss of H4K20me3 mediated by TGF-β/miR-29contributes to senescence and aging in a cellular-context and tissue-specific manner.

We also noted that increased H4K20me3 abundance was accompanied by down-regulated TGF-β signaling in *Zmpste24*-deficicent MEFs (Supplementary Fig. 10a–j). Considering that disruption of the nuclear periphery structure perturbs signal transduction^67^, depletion of Zmpste24 may negatively affect TGF-β-miR-29 signaling. Indeed, although the abundance of H4K20me3 was increased in *Zmpste24*-knockout cells in comparison with the same passage of wild-type cells, a global loss of H4K20me3 occurred during serial passages of *Zmpste24*-knockout cells (Supplementary Fig. 10k), and inhibition of TGF-β signaling led to elevated H4K20 methylation (Supplementary Fig. 10l, m) and decreased miR-29 expression (Supplementary Fig. 10n, o) in the late passages of wild-type and Zmpste24-depleted cells. These observations suggest that depletion of Zmpste24 blocks TGF-β-miR-29 signaling, which in turn results in elevated H4K20me3. The findings described above suggest that H4K20me3 is distinctively regulated in various senescent cell types and aging scenarios, which should be further explored in future studies.

Finally, we showed that E-616452, an inhibitor of TGF-β signaling, is capable of restoring the global H4K20me3 level in vivo and in vitro to establish a more “youthful” H4K20me3 signature. E-616452 is an essential compound in a cocktail used to chemically induce pluripotent stem cells^68^. Given that cellular senescence is not a limit to reprogramming^69,70^, and small molecule compounds can completely replace exogenous transgenes to generate pluripotent epigenetic and gene transcriptional profiles following somatic cell reprogramming, it appears possible to delay senescence or aging by targeting transcriptional networks and epigenetic features associated with cellular senescence using certain combinations of small-molecule compounds.

## Methods

### Generation of primary cells and cell culture

Primary mouse embryonic fibroblasts (MEFs) isolated from 12.5 to 14-day-old embryos of C57BL/6 mice, human embryonic fibroblasts (HEFs) isolated from 2- to 3-month-old human female embryos, and HEK293T from American Type Culture Collection (ATCC) were cultured in DEME (GIBCO, Grand Island, NY, USA), supplemented with 10% fetal bovine serum (FBS, GIBCO, Grand Island, NY, USA) and 1% Penicillin/Streptomycin (GIBCO, Grand Island, NY, USA), and incubated at 37 °C in a humidified incubator with 5% CO_2_. HEFs were obtained with informed written consent and approval by the Clinical Research Ethics Committee of China-Japan Friendship Hospital (No. 2009-50). The whole procedure was conducted according to the principles of the Declaration of Helsinki. The culture medium was MEM supplemented with 10% FBS (GIBCO), and the culture dish was gelatin-coated before inoculation of primary human umbilical vascular endothelial cells (HUVECs) purchased from ALLCELLS Co. Ltd., Shanghai, China. Cells were tested for mycoplasma contamination according to the manufacturer’s instructions (MycoBlue Mycoplasma Detector, D101-01/02).

To develop replicative senescence, confluent MEFs, HEFs, and HUVECs were evenly transferred into new dishes, after which the cells were cultured until confluence to generate one population doubling (PD). Cells were maintained in a 100-mm dish. In this study, confluent was defined as a nearly confluent culture condition without contact inhibition. The number (*n*) of PDs was calculated using the equation ${n = {\mathrm{log}}_2\,{\kern 1pt} {\mathrm{Ne/Ns}}}$, where Ne and Ns are the numbers of cells at the end of cell culture and those seeded at the start of one passage, respectively. The numbers of population doublings (PDs, times), doubling time (DT, days), and total culturing period were monitored once every three days until the cells completely stopped proliferating.

To isolate cardiomyocytes from adult mice, myocytes were separated in a high potassium buffer from the isolated heart tissue samples by modified Langendorff perfusion with type II collagenase. The hearts were removed from the anesthetized mice and subjected to cannulation, perfusion and digestion. Calcium was reintroduced into the dissociated cardiomyocytes, which were used in the following experiments.

### Senescence-associated β-galactosidase (SA-β-gal) staining

SA-β-gal activity, as a classic biomarker of senescent cells, was monitored for all MEFs growth stages of as described previously. The culture medium was removed from 6-well plates, after which the cells were washed once with 1 mL of 1× PBS and fixed with 0.5 mL of fixative solution for 10–15 min at room temperature. While the cells were in the fixative solution, the staining solution mixture (staining solution, staining supplement and 20 mg/mL X-gal in DMSO) was prepared according to the manufacturer’s instructions (Sigma, CS0030, USA). The cells were washed twice with 1 mL of 1× PBS and incubated overnight with the staining mixture at 37 °C. The cells were observed under an inverted microscope with 10 × 10 magnification (Leica DMI 6000B, Leica, Germany). The SA-β-gal signals were analyzed using Image J software (NIH).

### Real time and quantitative PCR (RT-qPCR)

Cells were harvested at 80% confluence. Total RNA was isolated with TRIzol Reagent (Invitrogen, 15596018, USA) according to the provided instructions. For RT-qPCR analysis, total RNA was reverse-transcribed into cDNA using random hexamer primers, and cDNA generated from mRNA, pri-miRNA and pre-miRNA levels was measured by qPCR (LightCycler, Roche, Swiss) with specific primers, after which the results were normalized using *Gapdh*. For mature miRNA, miRNA was reverse-transcribed according to the manufacturer’s instructions (Tiangen, KR211-02, China) and measured by qPCR with specific forward primers (see Supplementary Table 2) and commercially available reverse primers.

### Western blot

Total protein samples were resolved in 1% SDS, subjected to SDS-PAGE, transferred to nitrocellulose membranes and incubated overnight with primary antibodies of interest at 4 °C. The primary antibodies were anti-H3K4me3 (1:1000, Millipore, 04-745), anti-H3K9me1 (1:1000, Abcam, ab9045), anti-H3K9me2 (1:1000, Abcam, ab1220), anti-H3K9me3 (1:1000, Abcam, ab8898), anti-H3K9Ac (1:1000, Millipore, 07-352), anti-H3K27me3 (1:1000, Millipore, 07-449), anti-H3K36me2 (1:1000, Abcam, ab9049), anti-H3K36me3 (1:1000, Abcam, ab9050), anti-H4K16Ac (1:1000, Millipore, 07-329), anti-H4K20me1 (1:1000, Abcam, ab9051), anti-H4K20me2 (1:1000, Abcam, ab9052), anti-H4K20me3 (1:1000, Abcam, ab9053), anti-H3 (1:1000, Abcam, ab1791), anti-H4 (1:1000, Abcam, ab10158), anti-Suv4-20h1 (1:500, Abcam, ab118659), anti-Suv4-20h2 (1:500, Abcam, ab91224), anti-β-actin (1:2000, Santa Cruz, sc-47778), anti-p16 (1:1000, Santa Cruz, sc-1207), anti-p15 (1:500, Santa Cruz, sc-612), anti-p21 (1:500, Abcam, ab109199), anti-pSmad2 (1:1000, CST, #3101), anti-Smad2/3 (1:1000, CST, #3102), anti-Smad4 (1:1000, Abcam, ab40759), anti-γH2AX (1:1000, CST, #9718P), anti-Gapdh (1:2000, ZSGB-BIO, TA-08) and anti-α-tubulin (1:2000, Sigma, T9026). The blots were then washed three times in PBST (1× PBS and 0.1% Tween-20) and incubated with diluted secondary antibodies IRDye800CW Goat/Donkey anti-Mouse/Rabbit (1:10000, LI-COR, 926-32210) for 2 h at room temperature. Finally, the blots were washed three times with PBST and visualized using an Odyssey Infrared Imaging System (Odyssey, LI-COR).

Uncropped western blots can be found in Supplementary Figs. 11 and 12.

### Immunofluorescent microscopy

Cells were plated on clean cover slides in 100-mm culture dishes. When the cells reached 70–80% confluence, the cells on the slides were washed twice with ice-cold 1× PBS and fixed with 4% paraformaldehyde for 10 min at room temperature. The cells were washed three times with 1× PBS at room temperature, after which the cover slide was transferred to a humid dish. Next, 1% BSA was utilized to block the cells at room temperature for 30 min. The cells were washed cells three times with 1× PBS and incubated overnight at 4 °C with 150 μL of primary antibodies diluted in PBST. The primary antibodies were anti-Ki67 (1:400, Abcam, ab15580), anti-γH2AX (1:300, CST, #9718P), and anti-53BP1 (1:200, Abcam, ab36823). The cells were washed 4 times with PBST and incubated at room temperature for 2 h with 150 μL of secondary antibodies (1:500, Alexa Fluor 488 Donkey Anti-Mouse or Alexa Fluor 594 Donkey Anti-Rabbit, Life Technologies) diluted in PBST. The cells were washed cells three times with PBST and incubated with 1 ng/μL of DAPI at room temperature for 3 min. The cells were washed 3 times with 1× PBS, followed by a wash with ddH_2_O, after which the cover slide was sealed with 10 μL of Fluoromount-G. After 1 h, images of the cells were captured under a fluorescent microscope. The immunofluorescent signals were examined using Image J software (NIH).

### Luciferase reporter assay

For the luciferase assays of the miRNA targets, constructs were generated by inserting the full-length 3′ UTRs of mouse *Suv4-20h1* and *Suv4-20h2* into the pmirGLO vector (Promega, E1330) with specific primers. In total 50 nM of mimics and inhibitors of miRNAs were co-transfected with the constructs into HEK293T cells, respectively, using Lipofectamine 2000 (Invitrogen, 11668-027). Mimics and inhibitors of miR-29a and miR-29c were synthesized in RiboBio, China. In addition, the putative miR-29a and miR-29c seed sequence binding site (5′-TGGTGCT-3′) in the 3′ UTR of *Suv4-20h1* was mutated to 5′-CGCTACC-3′, meanwhile the two putative miR-29a and miR-29c seed sequence binding sites (5′-GTGCTAT-3′ and 5′-TGGTGCT-3′) in 3′ UTRs of *Suv4-20h2* were mutated to 5′-CTCCAAT-3′ (Mut-1), 5′-AGCTCCA-3′ (Mut-2) or both of these sequences. To test promoter activity utilizing luciferase assays, the predicted promoter regions of *Id1*, *p21*, *Mir29a* and *Mir29c* obtained from UCSC and NCBI were cloned into pGL3 (Promega) upstream of the luciferase gene. The empty or Smad4 overexpression vectors and recombinant pGL3 were co-transfected into HEK293T cells, and luciferase activity was monitored 24 h later. Luciferase activity was tested using the Dual-Glo Luciferase Assay Kit (Promega, E2920).

### Chromatin immunoprecipitation

Approximately 1.2 × 10^6^ MEFs were crosslinked with 1% paraformaldehyde for 10 min at room temperature. After quenching the paraformaldehyde reaction with 0.125 M glycine for 5 min, cells were harvested, lysed with nuclear lysis buffer (50 mM Tris-Cl, 10 mM EDTA, 1% SDS and protease inhibitors) on ice and sonicated with Bioruptor (Diagenode, Belgium) for the chromatin immunoprecipitation assays. Fragmented chromatin (25 μg) was subjected to immunoprecipitation with 4 μg of anti-Smad4 antibody (Abcam, ab40759) or anti-Rabbit IgG (Abcam, ab171870). The immunoprecipitated DNA was quantified by RT-qPCR. The ratio of DNA in the immunoprecipitates was calculated versus 10% input chromatin. The primer sequences used in this analysis are included in the Supplementary Table 2.

### Lentiviral production and viral transduction

To produce the lentiviruses of interest, HEK293T cells were transiently co-transfected with pLKO.1-shRNA encoding shRNAs targeting Suv4-20h1, Suv4-20h2 or Smad4 (TRC library, Sigma), as well as assistant vectors psPAX and pMD2.G. The culture medium was replaced with DMEM supplemented with 30% FBS 18 h later, and viral supernatants were collected 24 h later. Cells were infected with the packaged viruses with 5 μg/mL polybrene (M&C Gene Technology, MC032, China). 36–48 h after infection, cells were selected in 3 μg/mL of puromycin (M&C Gene Technology, MA009, China) for 24–36 h, after which they were plated into different dishes. Cells were then continuously selected in 1 μg/mL of puromycin for 4–5 days until no dead cells were found in the dishes.

### Nucleosomal DNA preparation

MEFs were collected in 1× PBS and resuspended in lysis buffer (15 mM Hepes, pH 7.4, 85 mM KCl and 0.5% NP-40) in the presence of a protease inhibitors cocktail (Roche) and PMSF, after which the nuclei were pelleted. The nuclei pellets were washed and resuspended in MNase digestion buffer (320 mM sucrose, 50 mM Tris·HCl, pH 7.5, 4 mM MgCl_2_, 1 mM CaCl_2_, 0.1 mM PMSF). To generate nucleosomal material, digestions were conducted by adding 0, 0.5 and 2 U of MNase per 0.1 mL of MNase digestion buffer. The reaction was incubated for 2 min at 37 °C and stopped by adding EGTA to a final concentration of 20 mM. The digested material was treated with RNase for 30 min at 37 °C, followed by Proteinase K treatment and two pheno-chloroform extractions. DNA was isolated from the extracted supernatant used to by adding with 3 M NaAc (pH 5.2) to a final concentration of 0.3 M Na^+^, 40 μg of glycogen and two-fold volumes of absolute ethanol. The precipitated DNA was analyzed by agarose gel electrophoresis and stained with ethidium bromide.

### Clonogenic cell survival assay

Wild-type and *SUV4-20H*-knockout cells (500 cells each) were plated separately in 100-mm culture dishes in triplicate and treated with etoposide at different final concentrations for 6 h, after which the medium was replaced with fresh medium without etoposide. Twelve days later, the cells were washed with 1×PBS, fixed with 4% formaldehyde for 15 min and stained with crystal violet (0.1% wt/vol) for 30 min. The number of colonies per dish was counted.

### Animal models

All animal experiments were approved by the Institutional Animal Care and Use Committee of Peking University. The mice used in this study were examined by the investigator in a double-blinded manner. When performing the echocardiographic imaging, the investigator lacked information regarding the treatment and was aware of only the strain and sex of the test mouse. Mice used in this study were not randomly chosen, and female and male mice were included.

### Statistical analysis

Approximately 100–300 cells were counted per sample, and three independent experiments were carried out. The data were analyzed by either a two-tailed unpaired Student’s *t*-test or one-way ANOVA with Dunnett’s multiple comparison test analysis (GraphPad Prism software, version 5.01). The results are expressed as mean ± s.d. Probability values (*p*) <0.05 were considered to be statistically significant.

### Data availability

All data supporting the findings in this study are available from the corresponding author upon reasonable request.

## Electronic supplementary material

Supplementary Information
