# Addendum: TGF-β signaling alters H4K20me3 status via miR-29 and contributes to cellular senescence and cardiac aging

**DOI:** 10.1038/s41467-018-06710-3

**Published:** 2018-10-08

**Authors:** Guoliang Lyu, Yiting Guan, Chao Zhang, Le Zong, Lei Sun, Xiaoke Huang, Li Huang, Lijun Zhang, Xiao-Li Tian, Zhongjun Zhou, Wei Tao

**Affiliations:** 1 The MOE Key Laboratory of Cell Proliferation and Differentiation, School of Life Sciences, Peking University, Beijing, 100871 China; 2 Department of Human Population Genetics, Human Aging Research Institute and School of Life Science, Nanchang University, Nanchang, 330031 China; 3 School of Biomedical Sciences, LKS Faculty of Medicine, The University of Hong Kong, 21 Sassoon Road, Hong Kong, China; 4 Shenzhen Institute of Innovation and Research, The University of Hong Kong, Nanshan, Shenzhen 518000 China

**Keywords:** Cell biology, Senescence

Addendum to: *Nature Communications* ; 10.1038/s41467-018-04994-z; published online 2 July 2018

Information relating to the in vivo treatments with A-196 and E-616452 was inadvertently omitted from the Methods section of this Article. In Fig. 6, Supplementary Fig. 7, as well as Supplementary Table 1, A-196 and E-616452 were used at a concentration of 25 mg/kg. A-196 and E-616452 were diluted in DMSO to a final concentration of 100 mM. Mice were treated with the inhibitors daily by oral gavage for 1 month.

