# Supplementary material for: TGF-β signaling alters H4K20me3 status via miR-29 and contributes to cellular senescence and cardiac aging
Source: Nat Commun. 2018 Jul 2;9:2560. doi: 10.1038/s41467-018-04994-z (PMC6028646; doi:10.1038/s41467-018-04994-z)
Supplement: Supplementary file 1 — Supplementary Information [file 41467_2018_4994_MOESM1_ESM.pdf]

TGF-β signaling alters H4K20me3 status via miR-29 and contributes to cellular senescence and cardiac aging

Guoliang et al.

Supplementary Figures

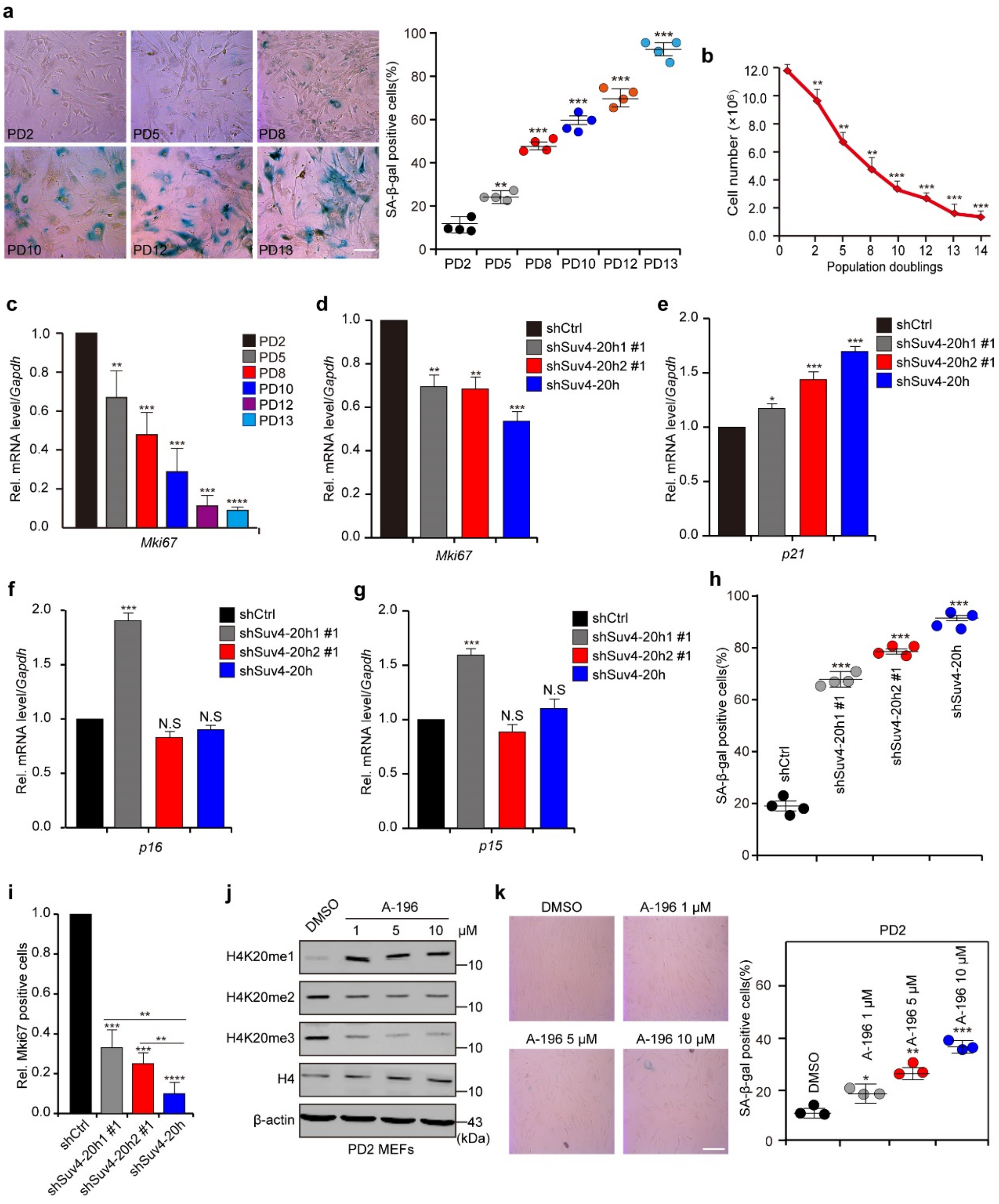

Supplementary Figure 1. Loss of H4K20me3 promotes emergence of senescent phenotypes.

a-c, MEFs progressively accumulated positive SA-β-gal stained cells and showed a decreased proliferation rate over time. MEFs from different growth stages were subjected to SA-β-gal staining (**a**), growth curve analysis (**b**) and *Mki67* measurement by RT-qPCR (**c**). Scale bar, 20 μm. **p<0.01, ***p<0.001 (One-way ANOVA with Dunnett's multiple comparison test). **d-g**, RT-qPCR measurements of Mki67 (**d**), p21

(**e**), p16 (**f**), and p15 (**g**) in Suv4-20h1 knockdown (shSuv4-20h1), Suv4-20h2 knockdown (shSuv4-20h2) or both (shSuv4-20h) cells. **h**, Statistical analysis of SA-β-gal staining in **Figure. 1i**. **i**, Statistical analysis of the Mki67 signal in **Figure. 1j**. **j**, Western blot for changes in H4K20 methylation in cells treated with the indicated doses of A-196. β-actin served as a loading control. **k**, Cells from **j** were subjected to SA-β-gal staining (left) and analyzed (right, One-way ANOVA with Dunnett's multiple comparison test). Scale bar, 20 μm. The error bars represent the s.d. obtained from triplicate independent experiments. Two-tailed unpaired Student's t-tests were performed. **$p<0.01$, ***$p<0.001$.

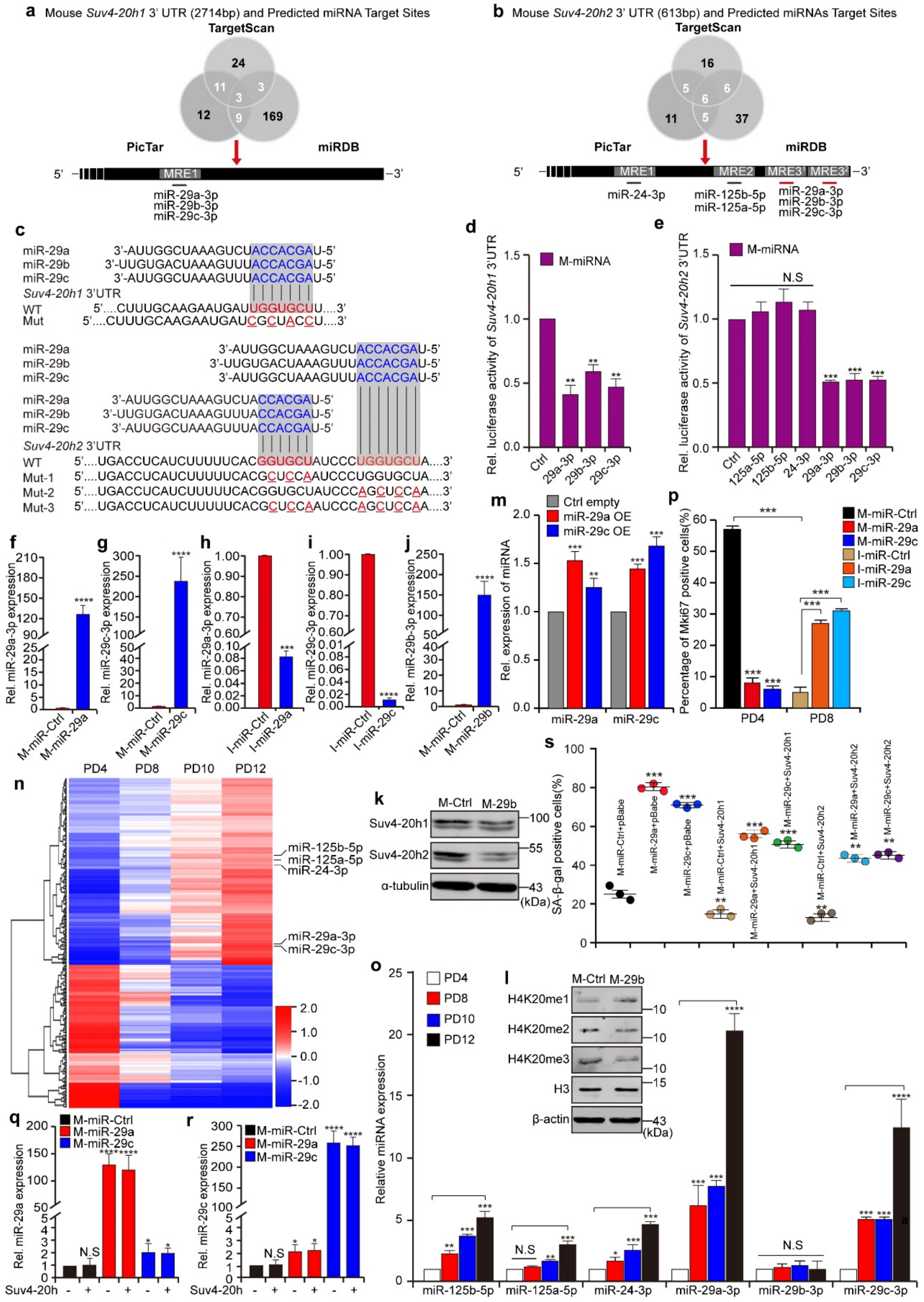

Supplementary Figure 2. miR-29 targets Suv4-20h to accelerate cellular senescence.

a, **b**, Schematic diagrams showing the of miRNAs predicted to target the 3' UTR of

Suv4-20h1 (**a**) or *Suv4-20h2* (**b**) by analyzing three different miRNA databases (TargetScan, PicTar and miRDB). **c**, Representative mode of miR-29 targeting the 3' UTRs of *Suv4-20h1* and *Suv4-20h2*. **d**, **e**, Luciferase activity assays showed cells co-transfected with miRNA mimics and constructs containing the 3' UTR of *Suv4-20h1* (**d**) or 3' UTR of *Suv4-20h2* (**e**). **f-j**, RT-qPCR showed expression of the indicated miRNA after transfection with the corresponding miRNA mimics or inhibitors. **k**, **l**, Western blots showed changes in Suv4-20h protein (**k**) and H4K20 methylation (**l**). α-tubulin and β-actin served as loading controls. **m**, RT-qPCR measurements of lentivirus-induced expression of miR-29. **n**, **o**, Differential miRNA profiling (**n**) and RT-qPCR measurement (**o**) of the indicated miRNAs during MEFs senescence. **p**, Statistical analysis of Mki67-positive cells from **Figure 2f,j**. **q**, **r**, RT-qPCR analysis of miR-29 expression in cells with mimic-mediated expression of miR-29 followed by reintroduction of Suv4-20h. **s**, Statistical analysis of SA-β-gal staining from **Figure 2i** (One-way ANOVA with Dunnett's multiple comparison test). The error bars represent the s.d. obtained from triplicate independent experiments. Two-tailed unpaired Student's t-tests were performed. $^{**}p<0.01$, $^{***}p<0.001$.

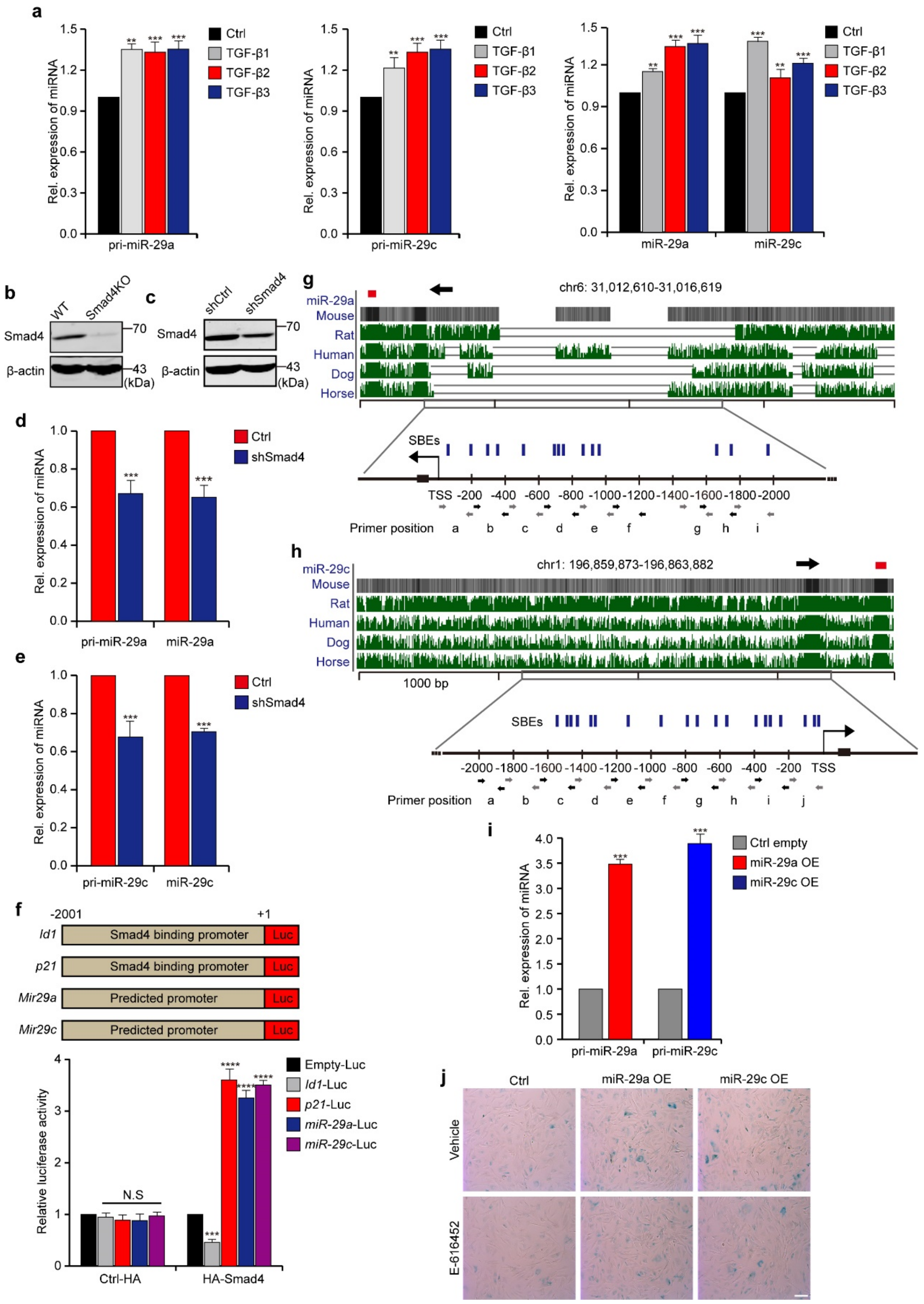

Supplementary Figure 3. TGF-β signaling regulates transcription of *Mir29* in a Smad-dependent manner during MEFs senescence.

a, RT-qPCR measurement of miR-29 expression in cells treated with or without TGF-

β1/β2/β3. **b**, **c**, Knockout (**b**) or knockdown (**c**) of Smad4 was confirmed by western blotting. **d**, **e**, RT-qPCR measurement of miR-29a (**d**) and miR-29c (**e**) upon Smad4 knockdown. **f**, Luciferase assays showed cells co-transfected with Smad4 and fused constructs containing the predicted promoters. **g**, **h**, Bioinformatics analysis of *Mir29a* (**g**) and *Mir29c* (**h**) about SBEs residing in the predicted promoters. The arrows show the transcription directions. The letters represent the primer positions for ChIP. **i**, RT-qPCR to measure miR-29 expression following lentivirus-mediated expression of miR-29. **j**, Cells with ectopic expression of miR-29 were subjected to SA-β-staining. The error bars represent the s.d. obtained from triplicate independent experiments. Two-tailed unpaired Student's t-tests were performed. $^{**}p<0.01$, $^{***}p<0.001$.

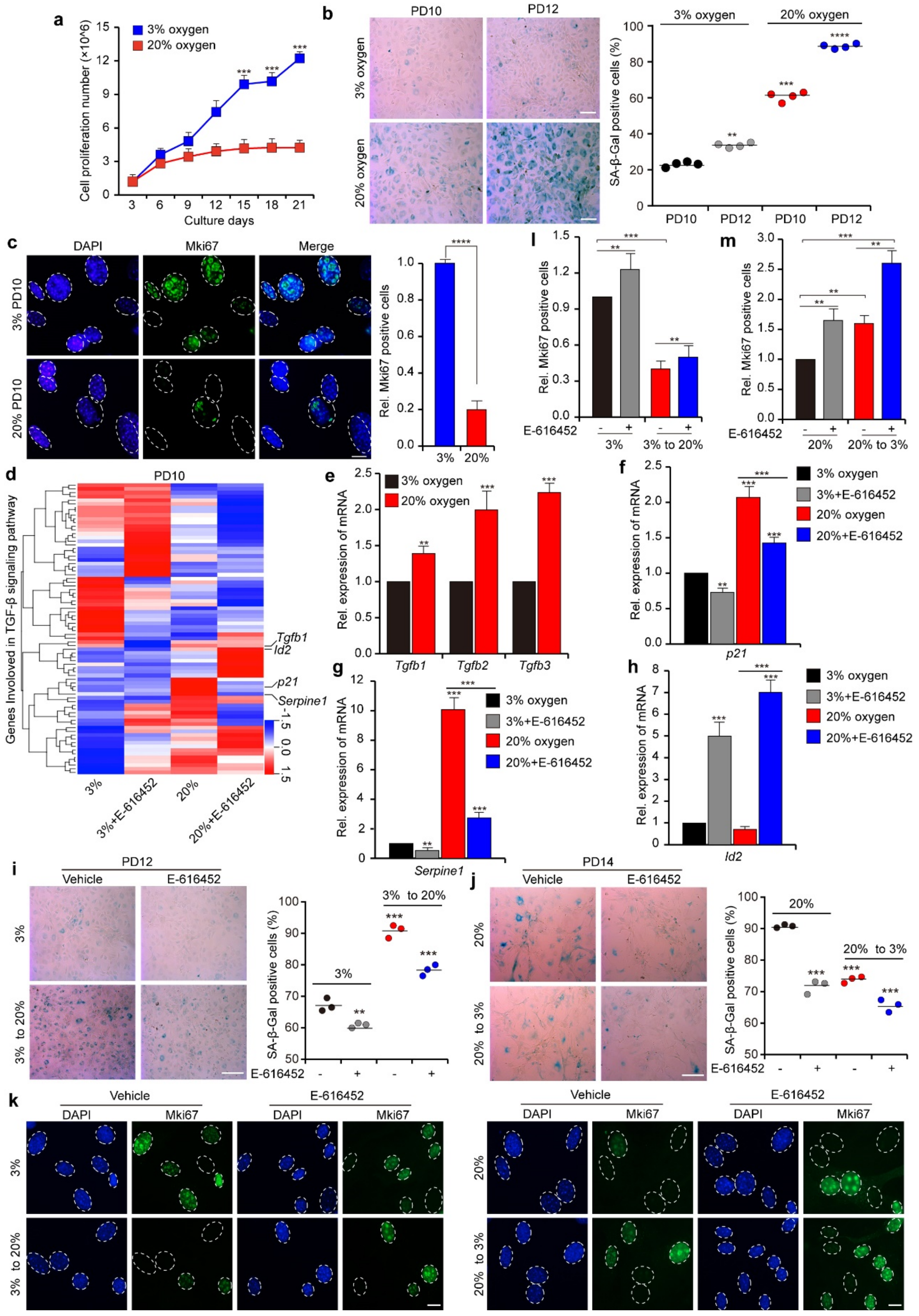

Supplementary Figure 4. Hyperoxia-induced oxidative stress activates TGF-β signaling to accelerate senescence.

a, The growth curves showed the differences in the proliferation rates of MEFs cultured under physioxia (3% oxygen) and relative hyperoxia (20% oxygen). **b**, Cells from PD10 and PD12 incubated in 3% or 20% oxygen were subjected to SA-β-gal staining. Scale bar, 20 μm. One-way ANOVA with Dunnett's multiple comparison test was performed. **c**, PD10 MEFs grown in 3% oxygen and 20% oxygen were used to immunofluorescent staining analysis of Mki67. Scale bar, 5 μm. Two-tailed unpaired Student's t-tests were performed. **d**, PD10 MEF cells grown in 3% or 20% oxygen, with or without inhibitors were collected for analysis of differential mRNA transcript expression. **e-h**, RT-qPCR measurement validated the mRNA expression results from **d**. **i**, **j**, MEFs grown in 3% oxygen and transferred to 20% oxygen (**i**), as well as MEFs cultured in 20% oxygen and transferred to 3% oxygen (**j**), were treated with or without inhibitors and subjected to SA-β-gal staining. Scale bar, 20 μm. One-way ANOVA with Dunnett's multiple comparison test was performed. **k-m**, Immunofluorescent staining analysis of the Mki67 signal of MEFs with the same treatment as in **j**. Graphs with p-values are representatives of triplicate independent experiments. $^{**}p<0.01$, $^{***}p<0.001$, $^{****}p<0.0001$.

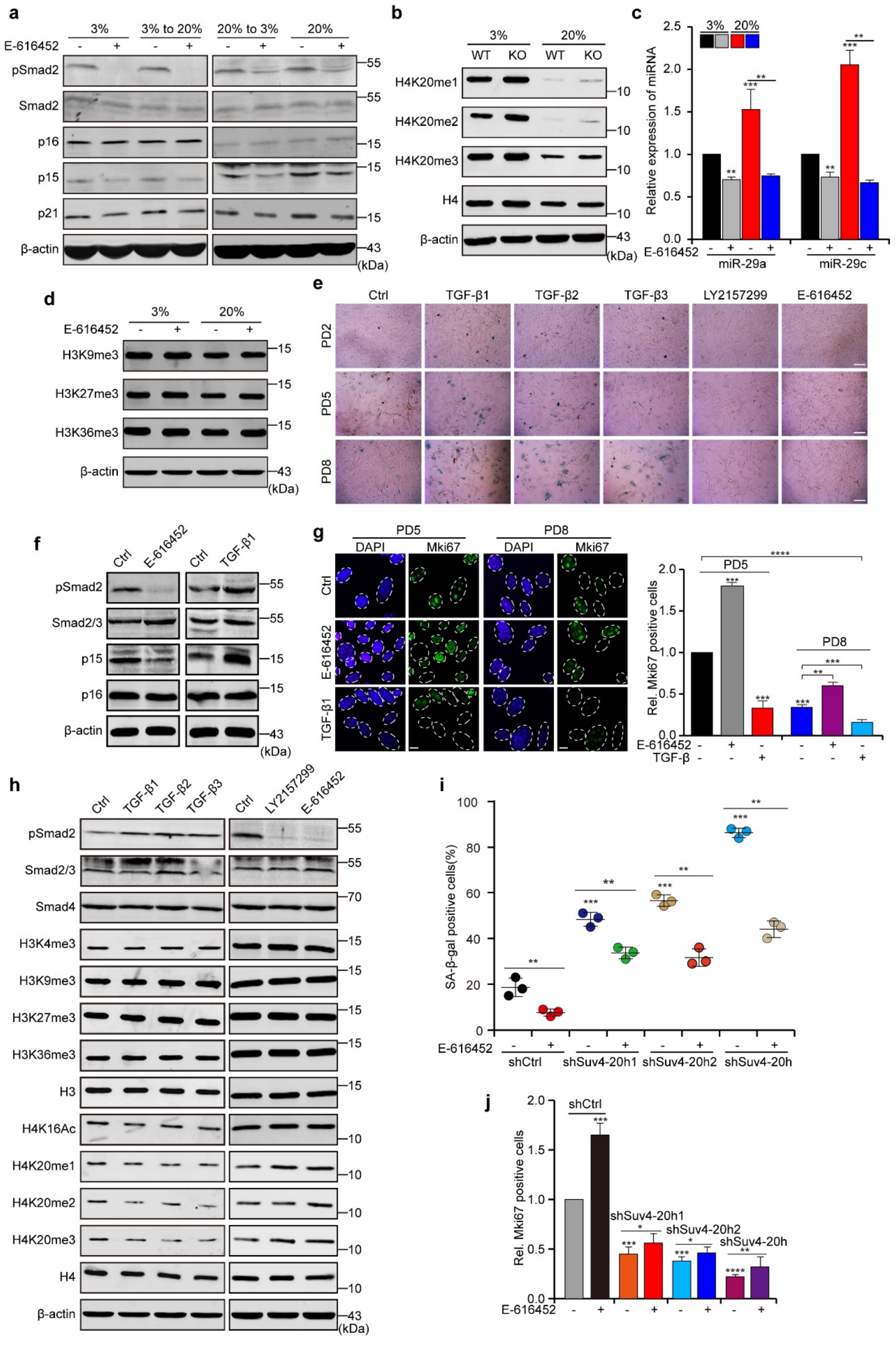

Supplementary Figure 5. TGF-β signaling regulates senescence by reducing

H4K20me3 abundance.

a, Western blots showed protein changes in MEFs grown in 3% or 20% oxygen. β-actin served as a loading control. **b**, Western blots showed changes in H4K20 methylation in MEFs grown in 3% or 20% oxygen upon Smad4 knockout (KO). WT is designated for wild-type. β-actin served as a loading control. **c**, RT-qPCR measurement of miR-29 expression in MEFs grown in 3% or 20% oxygen under treatment with or without E-616452. **d**, Western blot for alterations of histone modifications in cells cultured in 3% or 20% oxygen in the presence or absence of E-616452. β-actin served as a loading control. **e**, MEFs from PD2, PD5 and PD8 treated with TGF-β or inhibitors (LY2157299 and E-616452) were subjected to SA-β-gal staining. Scale bar, 20 μm. **f**, Western blot for confirmation of the efficacy of the treatments with E-616452 or TGF-β. **g**, Immunofluorescent microscopy for Mki67 staining in PD5 and PD8 MEFs (left) with the same treatment as in **f**. Signals were analyzed by ImageJ software (right). Student's t-test was applied. Scale bar, 5 μm. **h**, Western blot for the indicated proteins in cells with the same treatment as in **e**. β-actin served as a loading control. **i**, Statistical analysis of the SA-β-gal staining in **Figure 4g**. One-way ANOVA with Dunnett's multiple comparison test was performed. **j**, Statistical analysis of the Mki67-positive cells in **Figure 4h**. The error bars represent the s.d. obtained from triplicate independent experiments. Two-tailed unpaired Student's t-tests were performed. $^{**}p<0.01$, $^{***}p<0.001$.

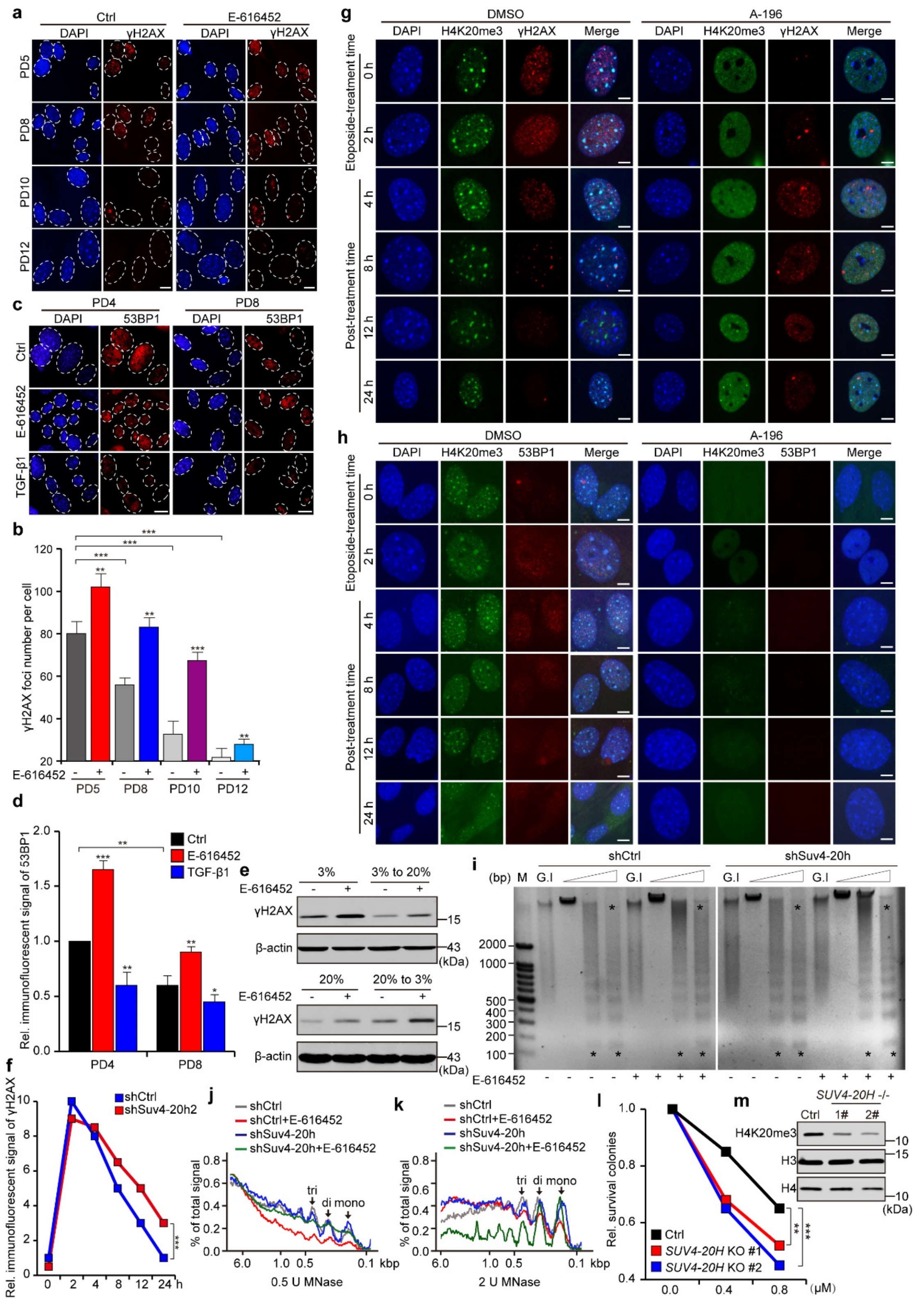

Supplementary Figure 6. Loss of H4K20me3 leads to DNA damage repair defects and lowers cell survival rates upon DNA damage stress.

a, **b**, Immunofluorescent staining of γH2AX in serially passaged cells treated with or without E-616452 (**a**). Signals were analyzed by using ImageJ software (**b**). Scale bar, 5 μm. **c**, **d**, Immunofluorescent staining of 53BP1 from PD4 and PD8 cells treated with inhibitor or TGF-β1 (**c**). Signals were analyzed using ImageJ software (**d**). Scale bar, 5 μm. **e**, Protein levels of γH2AX in cells treated with or without E-616452, were tested by western blotting. **f**, Statistical analysis of the γH2AX signals in **Figure 5e**. **g**, **h** Immunofluorescent staining for γH2AX (**g**) and 53BP1 (**h**) in cells incubated with A-196 for 48 h followed by etoposide treatment according to the indicated time course. Scale bar, 5 μm. **i-k**, Nuclei from Suv4-20h knockdown cells treated with or without E-616452 and incubated with 0 U, 0.5 U or 2 U of MNase for 5 min followed by DNA extraction, agarose gel electrophoresis and ethidium bromide staining. The asterisks indicate prominent signal changes (**i**). Signals were analyzed using ImageJ software (**j**, **k**). **l**, Colonies analysis of **Figure 5k** using ImageJ software. **m**, Western blot for H4K20me3 in HEK293T (Ctrl) and *SUV4-20H*-knockout cells (*SUV4-20H-/-* #1 and #2). H3 served as a loading control. The error bars represent the s.d. obtained from triplicate independent experiments. Two-tailed unpaired Student's t-tests were performed. $^{**}p<0.01$, $^{***}p<0.001$.

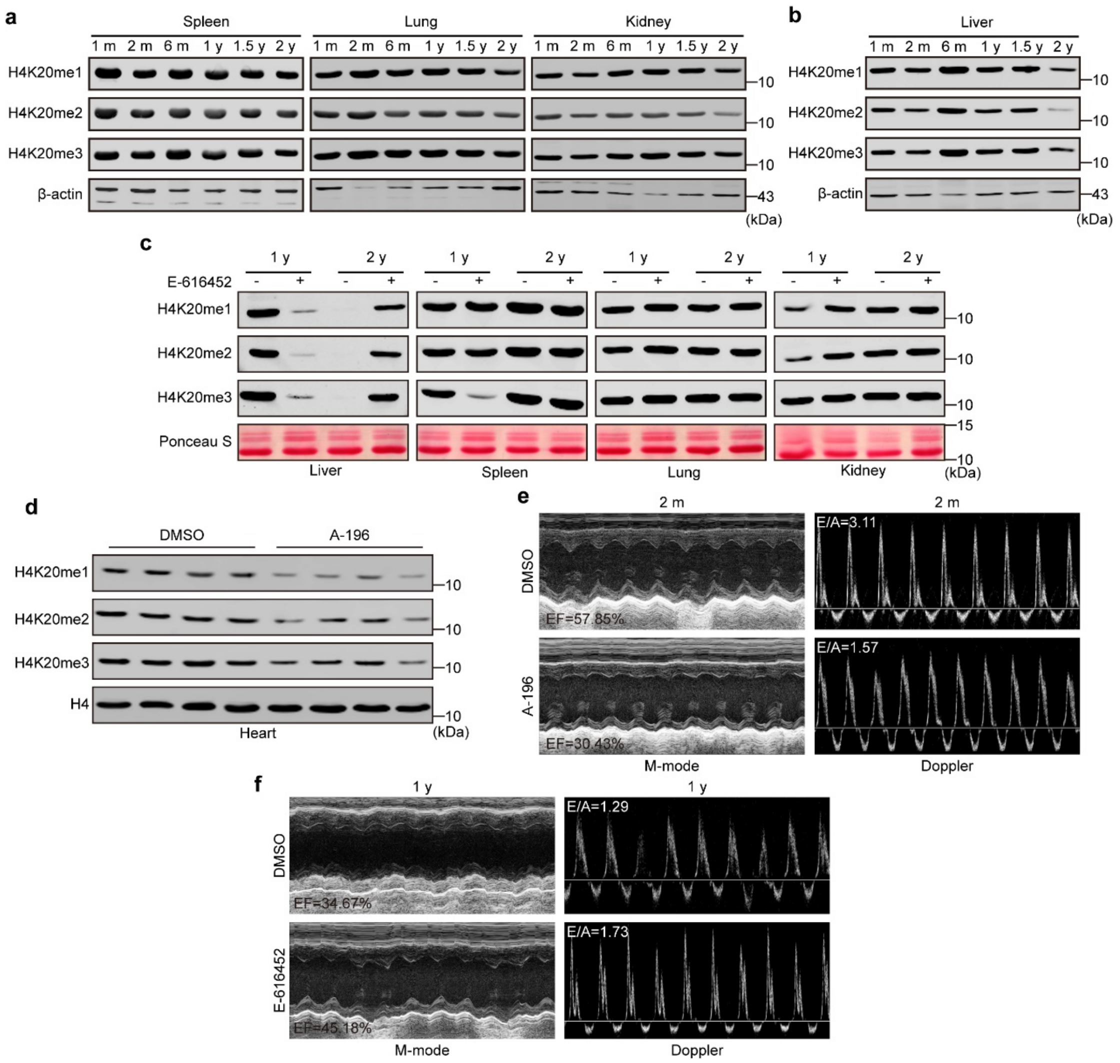

Supplementary Figure 7. TGF-β signaling regulates cardiac aging by reducing H4K20me3 abundance.

a, Tissues (spleen, lung and kidney) collected from mice of the indicated age were used to test H4K20 methylation by western blotting. **b**, Western blot for H4K20 methylation in livers collected from **a**. **c**, Immunoblotting for H4K20 methylation in livers, spleens, lungs and kidneys collected from the indicated mice fed with E-616452. H4, β-actin and Ponceau S served as loading controls. **d**, Western blot for H4K20 methylation in hearts from two-month-old mice. Four mice were treated with vehicle (DMSO), whereas four mice were treated with A-196. H4 served as a loading control. **e**, **f**, Representative echocardiographs vehicle (DMSO)- or A-196-treated 2-month-old mice (2m, **e**) and DMSO- or E-616452-treated 1-year-old mice (1 y, **f**). Data were acquired under B-mode, M-mode and pulse-waved Doppler.

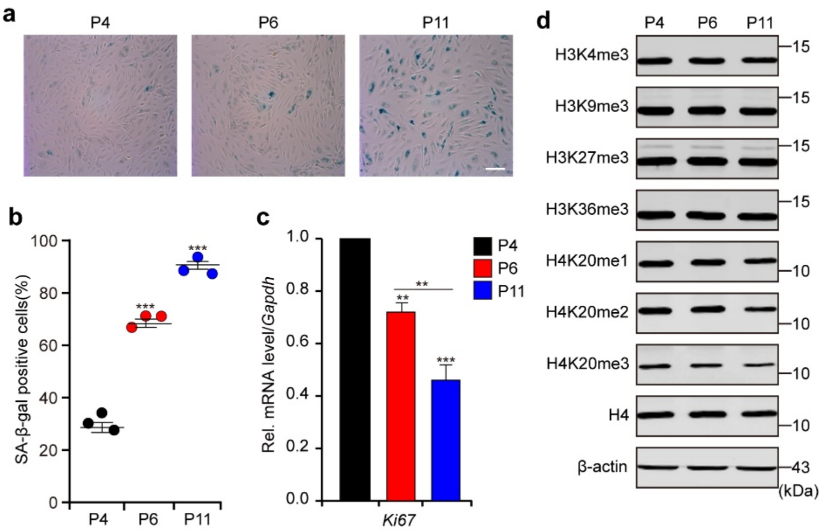

Supplementary Figure 8. H4K20 methylation is attenuated during HUVECs senescence.

a-c, HUVECs at different passage stages were subjected to SA-β-gal staining (**a**, **b**) and measurement of *Ki67* expression (**c**). Scale bar, 20 μm. One-way ANOVA with Dunnett's multiple comparison test was performed to analyze the SA-β-gal staining signals. **d**, The total protein levels of the indicated histone modifications were examined by immunoblotting. H4 and β-actin served as loading controls. The error bars represent the s.d. obtained from triplicate independent experiments. Two-tailed unpaired Student's t-tests were performed. $^{**}p<0.01$, $^{***}p<0.001$.

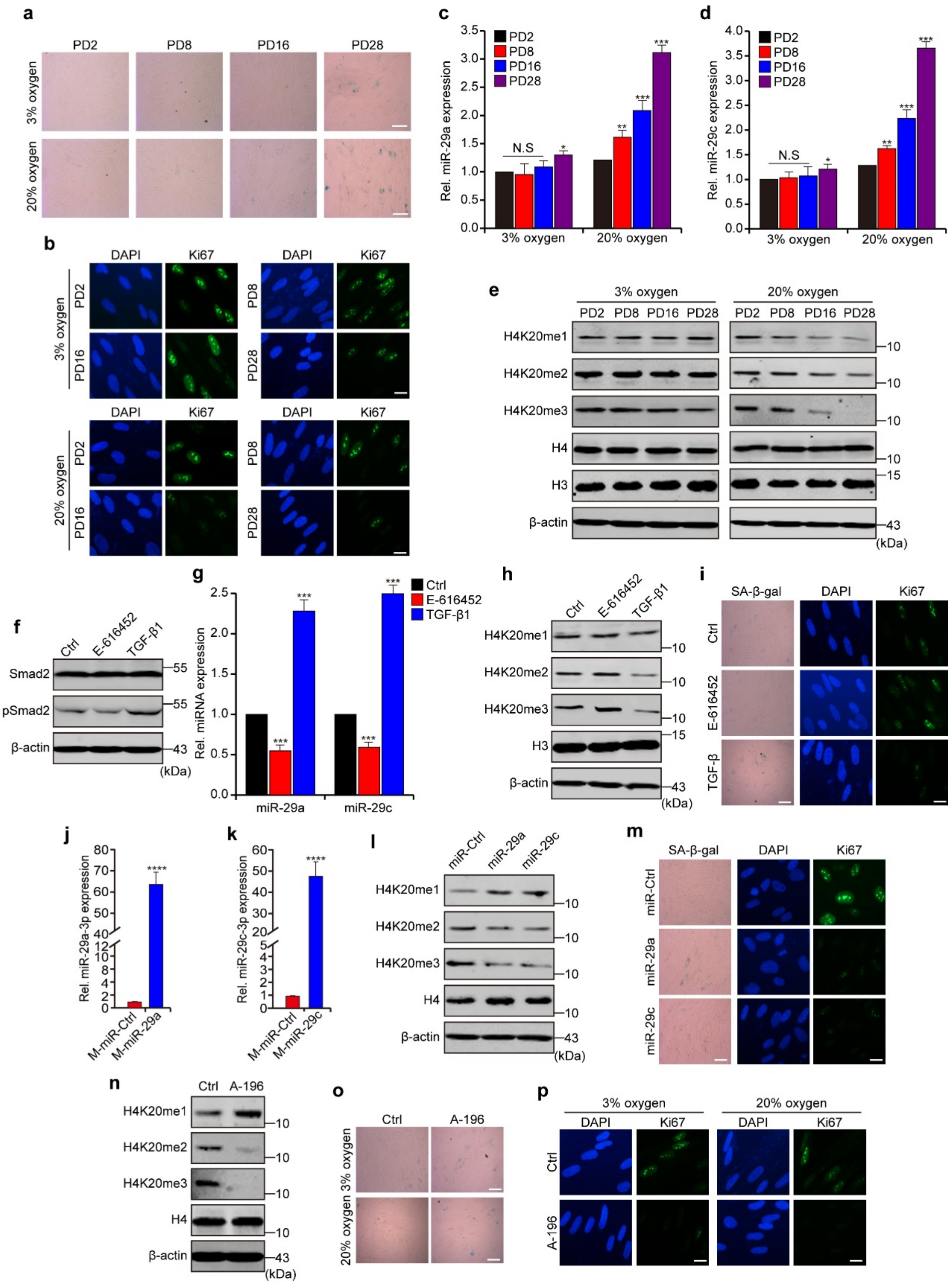

Supplementary Figure 9. Activated TGF-β signaling promotes HEFs senescence by reducing H4K20me3 via induction of miR-29 expression.

a, SA-β-gal staining of serially passaged HEFs (PD2, PD8, PD16 and PD28) individually grown in 3% and 20% oxygen. Scale bar, 20 μm. **b**, Immunofluorescent

staining of Ki67 of serially passaged HEFs cultured in 3% and 20% oxygen. Scale bar, 5 μm. **c**, **d**, RT-qPCR measurement of miR-29 expression in different passages of HEFs. miRNAs expression was normalized to U6. The error bar represents the s.d. from three independent replicates. *$p<0.05$, **$p<0.01$, ***$<p<0.001$, Student's t-test was performed. **e**, Global H4K20 methylation was tested by western blotting in the HEFs from **a**. H3, H4 and β-actin served as loading controls. **f**, Immunoblotting for the indicated proteins in the presence of E-616452 or TGF-β1. β-actin served as a loading control. **g**, RT-qPCR measurement of miR-29 expression in different passages of HEFs. **h**, Western blots of the indicated proteins in the HEFs derived from **f**. β-actin served as a loading control. **i**, SA-β-gal staining and immunofluorescent staining of Ki67 in HEFs with the same treatment as those in **f**. **j**, **k**, RT-qPCR measurement of miR-29 expression in HEFs with ectopic expression of miR-29 mimics. **l**, Western blot for total H4K20 methylation in HEFs with ectopic miR-29 expression. β-actin served as a loading control. **m**, SA-β-gal staining (left panel, scale bar, 20 μm) and immunofluorescent staining of Ki67 (right panel, scale bar, 20 μm) in HEFs with the same treatment as those in **l**. **n**, Immunoblotting of H4K20 methylation in cells treated with A-196. β-actin served as a loading control. **o**, **p**, SA-β-gal staining (**o**, scale bar, 20 μm) and immunofluorescent staining of Ki67 (**p**, scale bar, 5 μm) in cells with the same treatment as those in **a**.

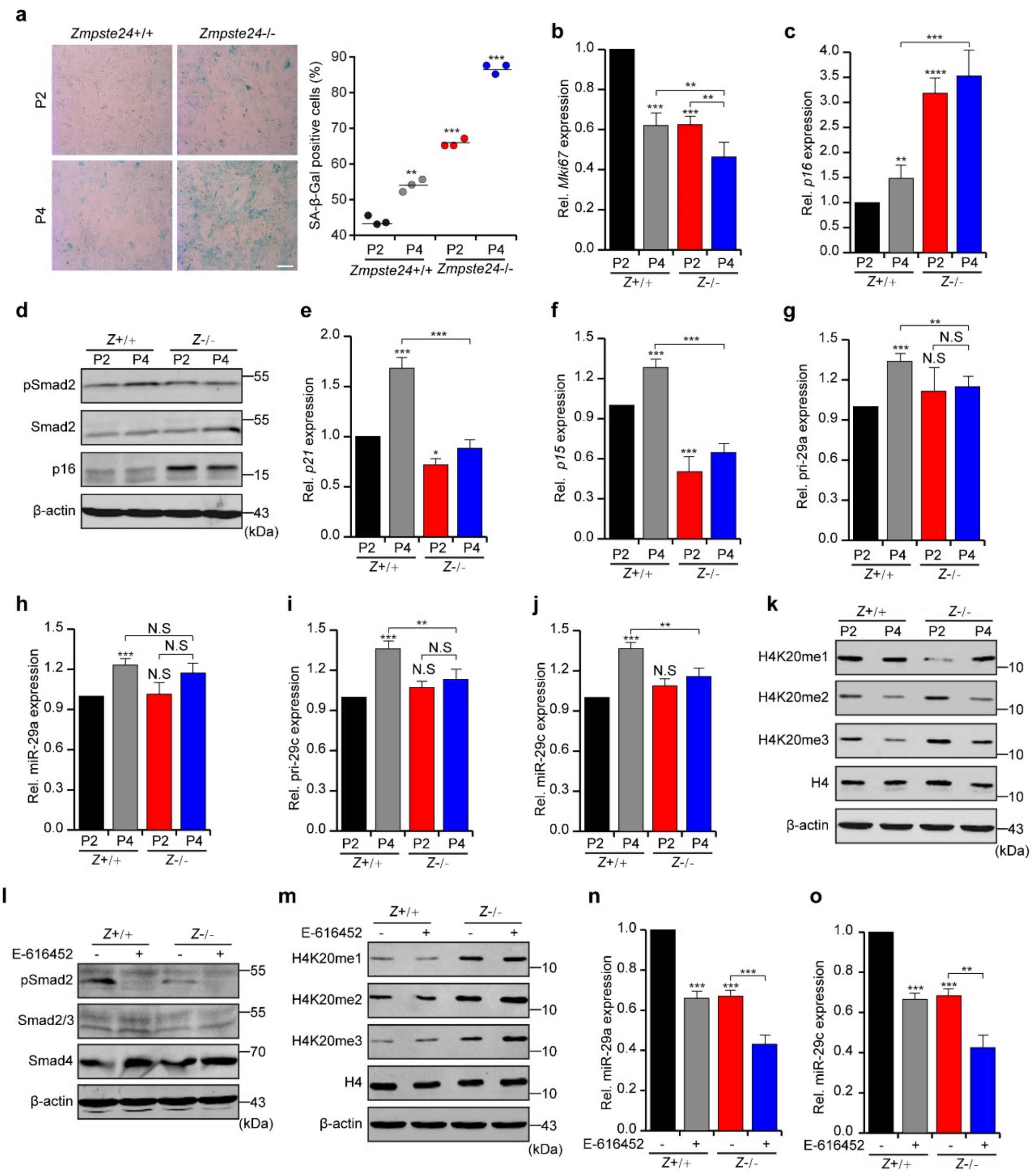

Supplementary Figure 10. Depletion of Zmpste24 blocks TGF-β-miR-29 signaling in the HGPS mouse model.

a, Serial passages (P2 and P4) of wild-type (*Zmpste24*$^{+/+}$) and *Zmpste24*-knockout (*Zmpste24*$^{-/-}$) MEFs were subjected to SA-β-gal staining (left panel). Signals were analyzed by ImageJ software. One-way ANOVA with Dunnett's multiple comparison test was performed (right panel). **b**, **c**, mRNA expression levels of *Mki67* (**b**) and *p16* (**c**) were tested by RT-qPCR and normalized to that of *Gapdh*. **d**, Total protein levels of pSmad2, Smad2 and p16 were examined by immunoblotting in serially passaged wild-

type and *Zmpste24*-knockout cells. β-actin served as a loading control. **e-j**, RT-qPCR measurement of the indicated genes (*p21*, **e** and *p15*, **f**) and miRNAs (pri-miR-29 and miR-29, **g-j**). Expression of mRNA and pri-miRNA was normalized to that of *Gapdh* and expression of miRNA was normalized to that of U6. Two-tailed unpaired Student's t-tests were performed. The error bars represent the s.d. obtained from triplicate independent experiments. **k**, Western blot for H4K20 methylation in the cells from **d**. β-actin served as a loading control. **l**, **m**, Immunoblotting of the indicated proteins in late-passage (P6) wild-type and *Zmpste24*-knockout MEFs treated with or without E-616452. β-actin and H4 served as loading controls. **n**, **o**, RT-qPCR measurement of miR-29a (**n**) and miR-29c (**o**) expression in cells from **l**. $Z^{+/+}$ and $Z^{-/-}$ represent wild-type and *Zmpste24* knockout cells in **b-o**, respectively. *p<0.05, **p<0.01, ***p<0.001.

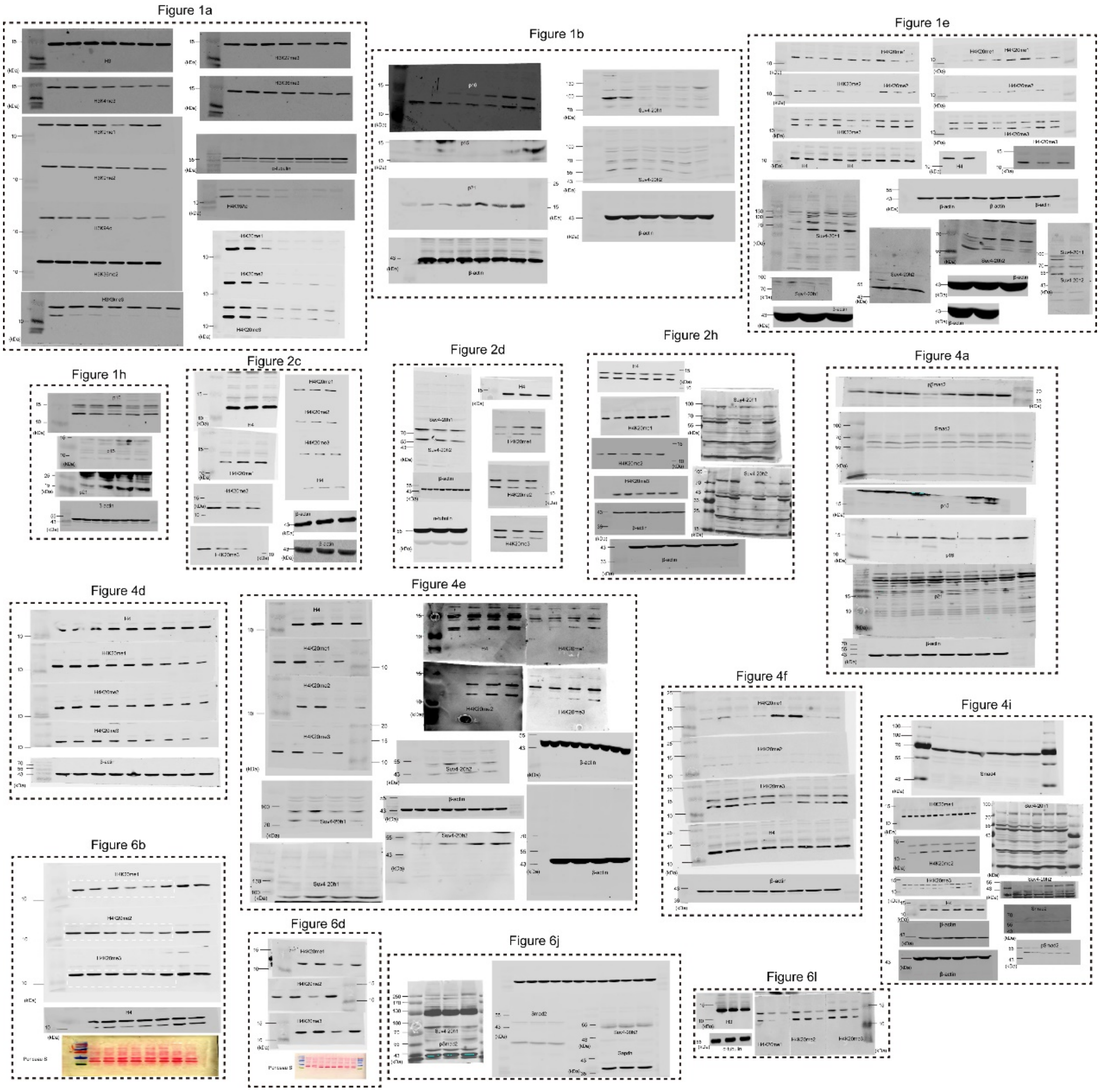

Supplementary Figure 11. Uncropped scans of Western blots from Fig. 1a, b, e, h; Fig. 2c, d, h; Fig. 4a, d, e, f, i; Fig. 6b, d, j, l

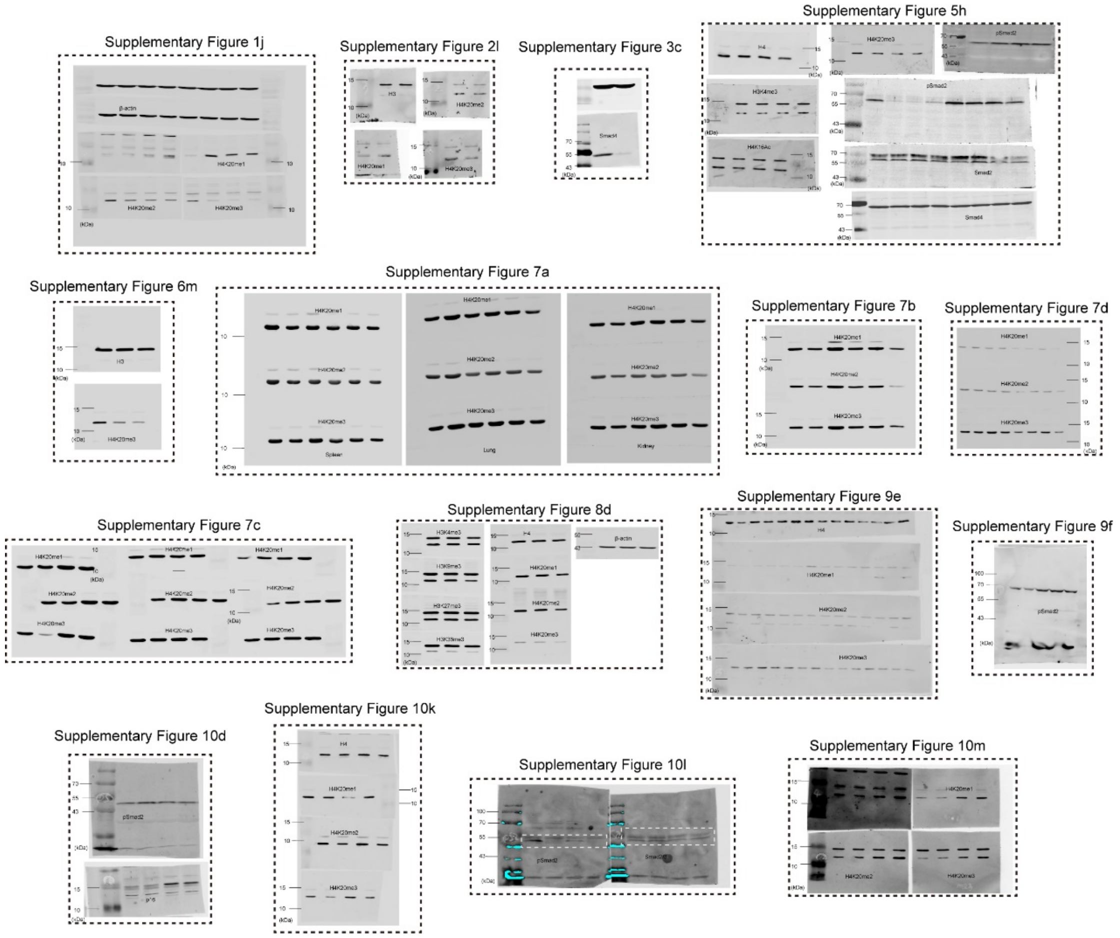

Supplementary Figure 12. Uncropped scans of Western blots from Supplementary Fig. 1j, Supplementary Fig. 2l; Supplementary Fig. 3b,c; Supplementary Fig. 5h, Supplementary Fig. 6m; Supplementary Fig. 7a, b, c, d; Supplementary Fig. 8d; Supplementary Fig. 9e, f; Supplementary Fig. 10d, k, l, m.

Supplementary Table 1 Diastolic and systolic data of left ventricle

	2 m (DMSO)	2 m (A -196)	1 y (DMSO)	1 y (E-616452)
LVAW; d	0.799 mm±0.054	0.632 mm±0.021	1.172 mm±0.027	1.385 mm±0.038
LVID; d	3.622 mm±0.115	4.128 mm±0.120	4.181 mm±0.160	3.956 mm±0.113
LVPW; d	0.826 mm±0.031	0.852 mm±0.043	0.959 mm±0.061	0.873 mm±0.057
LVAW; s	1.065 mm±0.041	1.092 mm±0.026	1.278 mm±0.016	1.225 mm±0.034
LVID; s	2.876 mm±0.053	4.234 mm±0.106	3.222 mm±0.082	2.476 mm±0.133
LVPW; s	0.692 mm±0.026	0.639 mm±0.017	0.985 mm±0.024	0.905 mm±0.027

Diastolic and systolic parameters of left ventricle under B- and M-mode. ± represents s.d. from mice of each group, respectively. Numbers of mice per group were indicated in the legend of **Figure 6e,f**. LVAWd, left ventricular anterior wall thickness at end-diastole; d, diastole; LVIDd, left ventricular internal dimension at end-diastole; LVPWd, left ventricular posterior wall thickness at end-diastole; LVAWs, left ventricular anterior wall thickness at end-systole; s, systole; LVIDs, left ventricular internal dimension at end-systole; LVPWs, left ventricular posterior wall thickness at end-systole.

Supplementary Table 2 Primers used in our study

Target	Forward (5′-3′)	Reverse (5′-3′)
qPCR *Mki67*	ATCATTGACCGCTCCTTTAGGT	GCTCGCCTTGATGGTTCCT
qPCR *Suv4-20h1*	ACTAGCGCCTTTCCTTCGAG	GCCGAAATCTCACAGGATTGTTG
qPCR *Suv4-20h2*	GCAGAGCTGCGTGAAGAGG	ACAGGCAGTATTCCCATCTGA
qPCR *p16*	CGCAGGTTCTTGGTCACTGT	TGTTCACGAAAGCCAGAGCG
qPCR *p21*	CCTGGTGATGTCCGACCTG	CCATGAGCGCATCGCAATC
qPCR *p15*	CCCTGCCACCCTTACCAGA	CAGATACCTCGCAATGTCACG
qPCR *Tgfb1*	GGCGGTGCTCGCTTTGT	GCGGGTGACCTCTTTAGCATAG
qPCR *Tgfb2*	CGGAGCGACGAGGAGTA	CGGACGATTCTGAAGTAGGGT
qPCR *Tgfb3*	GGACTGGCGGAGCACAA	CGCTGCTTGGCTATGTGC
qPCR *Serpine1*	CTTCAGCCCTTGCTTGCC	GGACCACCTGCTGAAACACT
qPCR *Ryr*	GCAGGTGGATGTGGAA	GTAGGAATGGCGTAGCA
qPCR *Myh7*	GAATGGCAAGACGGTGAC	TCCAGGAAGCGTAGCG
qPCR *Ki67*	GCCTGCTCGACCCTACAGA	GCTTGTCAACTGCGGTTGC
qPCR *Gapdh*	GTGTTCCTACCCCCAATGTGT	ATTGTCATACCAGGAAATGAGCTT
qPCR *GAPDH*	AGCCACATCGCTCAGACAC	GCCCAATACGACCAAATCC
qPCR Pri-miR-29a	AGCCCTGAAGTAAGTGTCC	AGGTCTTCATCCGAGCAT
qPCR Pri-miR-29c	ATGGGTGGAAGAGGGTT	TCTTCCAGGTTCAGACGAG
qPCR mmu-miR-29a-3p	GGCTAGCACCATCTGAAATCGGTTA	
qPCR mmu-miR-29c-3p	GGCTAGCACCATTTGAAATCGGTTA	
qPCR mmu-miR-29b-3p	GCGTAGCACCATTTGAAATCAGTGTT	
qPCR U6	CGCAAGGATGACACGCAAATTC	
ChIP miR-29a -414- -208	AACTATTGCACGGACTTCACCTTC	CTGGACACTTACTTCAGGGCTGTAC
ChIP miR-29a -621- -415	AGTAAAAAGTGTCACGCTTATCAAAA	CCTAATTTCAGGCGATCCTATG
ChIP miR-29a -828- -622	GCAGCAGTACTGATGATAGTGATAA	TCCAGATCGATCTGTTAGAGTCG
ChIP miR-29a -1035- -829)	TTATTGGAGTCCCTGACACATTC	TACTGCTACTACTATTACAGTTTTAAA
ChIP miR-29a -1236- -1021	GATTTGAGAATGAGCAGGAA	CAGGGACTCCAATAACTACAC
ChIP miR-29a -1656- -1450	GTCCCATGCACACTGGCTACTTC	GTTCTTACTGAATTGTTTGAAAGCA
ChIP miR-29a -1780- -1559	AATAGATTCCCAGTCTGTAGC	CTACCCGAGGAAACAAAC
ChIP miR-29a -2001- -1752	CCGAGCTCATTCTCCAGCCC	GGTGCCAGGCTACAGACTGGG
ChIP miR-29c -207- -1	GGGTTCCTTGGGCCTGCAC	GGACCGACTGGTGGTGTTCTTC

ChIP miR-29c -414- -208	CACAGCAGAGGGTAGACTACAGAGG	TCTTCCACCCATGGAATGCTG
ChIP miR-29c -621- -415	GCTCAAAGTGTTGGCTGTATG	CTGCAATTCTTACTCCTAAAACAC
ChIP miR-29c -828- -622	GTTGTTAACATCTCATAGGTCATTG	AGACAAAAACAATGCAAAGCTT
ChIP miR-29c -1035- -829	AGGTACATTTTTAAAACATACCTTGTT	TGAACCCAAGTAAGTCACCTGTG
ChIP miR-29c -1242- -1036	TCACATTATATTTGCTCTATTACCC	AAAAATAAGATTCACAAGCTATGTT
ChIP miR-29c -1449- -1243	CCCCTCCCCAGTCTAGCTCT	GTATCCCCAGAGTCACAATATTCAA
ChIP miR-29c -1656- -1450	GAGAAGTCAGTGTCAGCATTGCTAG	GATTTAGCTCAGACAATCTGGATGG
ChIP miR-29c -1863- -1657	ACTGCCATCTAATCTATTTATGACC	AGAAACTGGGCCAGCCA
ChIP miR-29c -2001- -1864	CATAGAGGGAGGGGATTGG	TATTAACAAGGGTCAAAATCCTG
Promoter miR-29a-3p	GGGGTACCCCGAGCTCATTCTCCAGCC	CGCTCGAGCACAAGAGGTCATGTGCA
Promoter miR-29c-3p	GGGGTACCCATAGAGGGAGGGGATTG	CGCTCGAGGGACCGACTGGTGGTGTT
3′ UTR-*Suv420h1*	GAGCTCTTGAATCTTGTGCGTGAC	GTCGACCTCTTTACATTGAATCCC
3′ UTR-*Suv420h1*-Mut	CAAGAATGATCGCTACCTATTTTTC	TAGGTAGCGATCATTCTTGCAAAG
3′ UTR-*Suv4-20h2*	CGAGCTCCTTACAGGGAGCGGAATG	GCGTCGACCCAGTAGCACCAGGGATA
3′ UTR-*Suv4-20h2*-Mut	GAGCTCCTTACAGGGAGCGGAATGGA	
3′ UTR-*Suv4-20h2*-Mut-1		GTCGACCCAGTTGGAGCTGGGATAGC
3′ UTR-*Suv4-20h2*-Mut-2		GTCGACCCAGTAGCACCAGGGATTGG
